# CtBP1/2 differentially regulate genomic stability and DNA repair pathway in high-grade serous ovarian cancer cell

**DOI:** 10.1038/s41389-021-00344-9

**Published:** 2021-07-13

**Authors:** YingYing He, Zhicheng He, Jian Lin, Cheng Chen, Yuanzhi Chen, Shubai Liu

**Affiliations:** 1 School of Chemical Science & Technology Yunnan University Kunming, Yunnan, 650091 China; 2 State Key Laboratory of Phytochemistry and Plant Resources in West China Kunming Institute of Botany, Chinese Academy of Sciences Kunming, Yunnan, 650201 PR China; 3 University of Chinese Academy of Sciences, Beijing, 100049 China

**Keywords:** Cancer genetics, Apoptosis, DNA damage response, Double-strand DNA breaks

## Abstract

The C-terminal binding proteins (CtBPs), CtBP1 and CtBP2, are transcriptional co-repressor that interacts with multiple transcriptional factors to modulate the stability of chromatin. CtBP proteins were identified with overexpression in the high-grade serous ovarian carcinoma (HGSOC). However, little is known about CtBP proteins’ regulatory roles in genomic stability and DNA repair in HGSOC. In this study, we combined whole-transcriptome analysis with multiple research methods to investigate the role of CtBP1/2 in genomic stability. Several key functional pathways were significantly enriched through whole transcription profile analysis of CtBP1/2 knockdown SKOV3 cells, including DNA damage repair, apoptosis, and cell cycle. CtBP1/2 knockdown induced cancer cell apoptosis, increased genetic instability, and enhanced the sensitivity to DNA damage agents, such as γ-irradiation and chemotherapy drug (Carboplatin and etoposide). The results of DNA fiber assay revealed that CtBP1/2 contribute differentially to the integrity of DNA replication track and stability of DNA replication recovery. CtBP1 protects the integrity of stalled forks under metabolic stress condition during prolonged periods of replication, whereas CtBP2 acts a dominant role in stability of DNA replication recovery. Furthermore, CtBP1/2 knockdown shifted the DSBs repair pathway from homologous recombination (HR) to non-homologous end joining (NHEJ) and activated DNA-PK in SKOV3 cells. Interesting, blast through TCGA tumor cases, patients with CtBP2 genetic alternation had a significantly longer overall survival time than unaltered patients. Together, these results revealed that CtBP1/2 play a different regulatory role in genomic stability and DSBs repair pathway bias in serous ovarian cancer cells. It is possible to generate novel potential targeted therapy strategy and translational application for serous ovarian carcinoma patients with a predictable better clinical outcome.

## Introduction

Serous ovarian cancer is a gynecological tumor that is more common in women, and high-grade serous ovarian cancer (HGSOC) accounts for roughly 70% of ovarian cancer deaths [1, 2]. As an aggressive tumor type of ovarian cancer, HGSOC had a higher relapse rate (~25%) and poorer overall 5-year survival rate (31%) [3, 4]. The genetic alterations of HGSOC were well characterized, including copying number gains and losses, mutations and deletions [5], and were associated with a homologous recombination (HR) defect and increased sensitivity to DNA damage agents [5, 6]. Intracellular signaling is activated by genetic alterations to detect mispatch and regulate cell cycle progression and promote the repair of DNA lesions by DNA damage response (DDR). Double-strand breaks (DSBs) are among the most lethal types of DNA lesions in mammalian cells, and they are primarily repaired by the non-homologous end joining (NHEJ) or HR pathways. Pathways for DNA repair disruption, whether through chemotherapy drugs or other clinical cancer treatment methods, had been identified as an effective therapeutic strategy for HGSOC [7, 8].

The C-terminal binding protein (CtBP) family proteins consisted of two isoforms, CtBP1 and CtBP2, that shared a highly conserved protein structure (78% sequence homolog) and performed some similar functions in human cells, such as carbohydrate metabolism and epigenetic regulatory multiple transcription factors [9]. CtBP proteins had a distinct intracellular distribution that was determined by a C-terminal PDZ binding motif (CtBP1) and a unique N-terminal nuclear localization domain (CtBP2). CtBP1 was found in both the cytoplasm and the nucleus, whereas CtBP2 was exclusively resident in the nucleus [10]. CtBP1 interacted with human adenovirus E1A via a PLDLS motif and would be functional as a tumor suppressor [11, 12]. CtBP proteins had been found to be overexpressed in a variety of solid tumors, including breast, ovarian, prostate, colon, and gastric cancer, and had been linked to poor patient survival in clinical outcome [13, 14]. CtBP2 had been identified as a novel oncogene in serous ovarian cancer [15, 16], and overexpression of CtBP2 had been linked to abnormal proliferation and a lower survival rate [13]. CtBP proteins suppressed the expression of death receptors D4/5 and determine the fate of serous ovarian cancer cells [17]. CtBP proteins inhibited apoptosis by decreasing the expression of pro-apoptotic genes [18–20]. Up to now, little is known about the contribution of CtBP proteins in genomic stability in serous ovarian cancer.

In this study, we combined whole-transcriptome analysis with multiple DNA damage repair research methods to investigate the role of CtBP1/2 in genomic stability in serous ovarian cancer cells. Several enriched key functional pathways, including DDR and apoptosis, had been thoroughly investigated in CtBP1/2 knockdown serous ovarian cancer cells utilizing transcription profile enrichment analysis. CtBP proteins had been studied for their role in genomic stability and DNA repair.

## Results

### Established CtBP1/2 stable knockdown in serous ovarian cancer cells

**Fig. 1:**
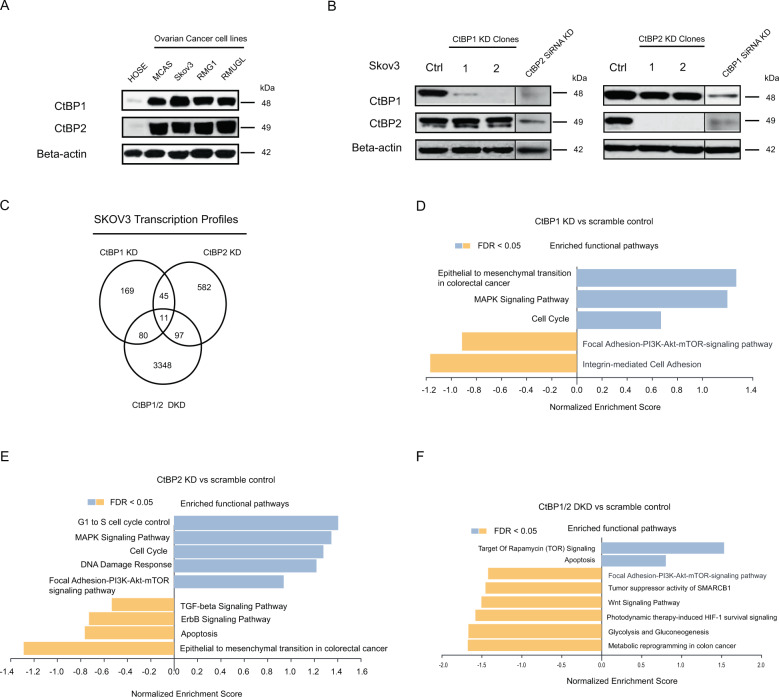
CtBP1/2 overexpressed in ovarian cancer cell lines and stable knockdown cells transcription profiling analysis. The overexpression of CtBP1/2 was analyzed by western blot in ovarian cancer cell lines (**A**). CtBP1/2 stable knockdown in SKOV3 cells generated by lentivirus containing CtBP1/2-targeting shRNA constructs and siRNA transient double knockdown (KD) (**B**). The Venn diagram showed the overlapped significant changed genes among CtBP1KD, CtBP2 KD, and CtBP1/2 double KD (**C**). The enriched functional pathways were summarized in CtBP1KD vs. control (**D**), CtBP2 KD vs. control (**E**), and CtBP1/2 double KD vs. control (**F**).

CtBP1/2 was found to be abnormally overexpressed in several ovarian cancer cell lines, including MCAS, SKOV3, RMG1, and RMUGL (Fig. 1A), and a very weak signal was found in human normal ovarian epithelium (HOSE). It is thought that CtBP1/2 overexpression contributes to the abnormality of ovarian cancer cells. The lentivirus shRNA constructs successfully generated scramble control and CtBP1/2 knockdown (CtBP1 KD, CtBP2 KD) SKOV3 cells. To avoid off-target effects, each target gene was initially screened with five different shRNA constructs, and then selected two independent clones for the next step functional assay, which were validated by Western blot with clearly CtBP knockdown (Fig. 1B). Similar to our previous findings [21], specific single knockdown of the CtBP1 or CtBP2 gene did not significantly induce isoform gene compensatory expression. Transiently dual CtBP1/2 knockdown had been achieved by a 48-h swap of CtBP1/2 siRNA interference in CtBP1/2 stable knockdown cancer cells, and validated by western blot analysis (Fig. 1B).

### Transcriptome analysis discovered key functions regulated by CtBP1/2 proteins

Normalized and filtered through comparison, 355 genes (upregulated 182 genes; downregulated 173 genes; CtBP1 KD vs. control, Fig. S1A), 805 genes (upregulated 784 genes; downregulated 21 genes; CtBP2 KD vs. control, Fig. S1B) and 4297 genes (upregulated 1364 genes; downregulated 2933 genes; CtBP1/2 DKD vs. control, Fig. S1C) had been identified as significantly changed genes and computationally clustered, respectively. A Venn diagram demonstrated the overlapped significantly changed genes among three knockdown groups (Fig. 1C). Different groups shared a variety of common genes, including 56 genes (CtBP1 KD vs. CtBP2-KD) (Table S1), 91 genes (CtBP1 KD vs CtBP1/2 DKD), 108 genes (CtBP2 KD vs CtBP1/2 DKD), and 11 genes were overlapped for three groups. Key functional pathways were enriched and characterized in CtBP1 KD, CtBP2 KD, and CtBP1/2 DKD cells using GSEA. Three key pathways were negatively enriched in CtBP1-KD cells, including epithelial to mesenchymal transition in colorectal cancer, MAPK signaling pathway, and cell cycle, while focal adhesion-PI3K-Akt-mTOR-signaling and integrin-mediated cell adhesion were positively enriched (Fig. 1D). Positively enriched pathways in CtBP2-KD cells included G1 to S cell cycle control, MAPK signaling pathway, cell cycle, DDR, and focal adhesion-PI3K-Akt-mTOR-signaling pathway; negatively enriched pathways included TGF-beta signaling pathway, ErbB signaling pathway, apoptosis, and epithelial to mesenchymal transition in colorectal cancer (Fig. 1E). Positively enriched pathways in CtBP1/2-DKD cells included rapamycin (TOR) signaling and apoptosis target; negatively enriched pathways included focal adhesion-PI3K-Akt-mTOR-signaling pathway, tumor suppression activity of SMARCB1, Wnt signaling pathway, photodynamic therapy-induced HIF-1 survival signaling, and glycolysis and gluconeogenesis, and metabolic reprogramming in the colon cancer (Fig. 1E). Cell cycle and focal adhesion-PI3K-Akt-mTOR signaling pathways were common enriched pathways between CtBP1/2-KD cells. Overexpression of CtBP1/2 was thought to play regulatory roles in the cell cycle and adhesion of serous ovarian cancer cells. The apoptosis pathway, in particular, had been highlighted in the enriched functional pathways of CtBP1/2-DKD and CtBP2-KD cells.

### CtBP1/2 knockdown impaired cellular functions of serous ovarian cancer cell

Because the epithelial to mesenchymal transition and adhesion-related pathways were enriched in the CtBP1-KD/CtBP2-KD cells, the ability of cell proliferation, adhesion and migration were evaluated. CtBP protein knockdown significantly reduced cell proliferation (Fig. S2A), clone formation in vitro agar gel (Fig. S2B), and adhesion to extracellular matrix proteins collagen I and collagen IV (Fig. S2C), as measured by the scratch wound and transwell assays (Fig. S2D&E). CtBP2-KD had a greater impact on cellular functions, according to these findings. CtBP2 inhibition is proposed as an appropriate therapeutic target for serous ovarian cancer therapy and as an effective treatment strategy for serous ovarian cancer.

### CtBP1/2 knockdown triggered apoptosis in serous ovarian cancer cells

**Fig. 2:**
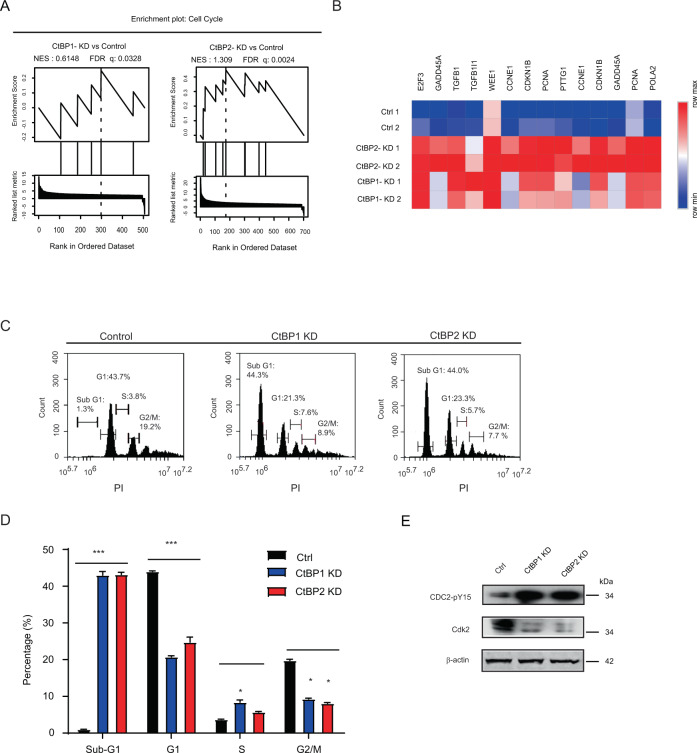
CtBP1/2 knockdown impacted cell cycle of ovarian cancer cells. The enrichment plot of cell cycle-related genes was identified by GSEA in CtBP1 KD (left) and CtBP2 KD (right), compared to control cells (**A**). Heat maps significantly comparing the expression of genes that involved in cell cycle regulation in CtBP1/2 KD and control groups (**B**). The comparison between CtBP1/2 KD and control groups in cell cycle by flow cytometry (**C**, **D**). The cell cycle profile also was created by measuring total DNA content with PI. Data were representing each phase of cell cycle and quantitative analysis the percentage generated by three independent experiments. Western blot analyzed the changed pattern of cell cycle key regulators between CtBP1/2 knockdown and control (**E**). All the data represented the average of triplicates values generated by three independent experiments. *P < 0.05; **P < 0.001; ***P < 0.0001.

Because the cell cycle signaling pathway was significantly positively enriched in CtBP1-KD/CtBP2-KD cells (Fig. 1D, E), cell cycle-related genes were gathered for further analysis (Fig. 2A). The heatmap depicts the 14 genes involved in cell cycle regulation that were significantly upregulated in CtBP1-KD and CtBP2-KD cells (Fig. 2B). Knockdown CtBP1 or CtBP2 disrupted the cell cycle phases, significantly increased the portion of sub-G1 phase (CtBP1-KD, 44.3%; CtBP2-KD, 44.0%), and decreased the portion of G1 phase and G2/M phase (Fig. 2C, D). It is proposed that CtBP1 or CtBP2 knockdown induce apoptosis in serous ovarian cancer cells, and that CtBP1/2-DKD accelerate the process of apoptosis while failing to generate stable double knockdown cells. In CtBP1-KD/ CtBP2-KD cells, the phosphorylation level of Cdc25A-Y15 increased while the expression level of Cdk2 decreased (Fig. 2E), both of which were indicators of cell cycle progression and enhanced apoptosis [22].

### CtBP1/2 knockdown exacerbated the DNA damage response to irradiation and chemotherapy drugs in serous ovarian cancer cells

**Fig. 3:**
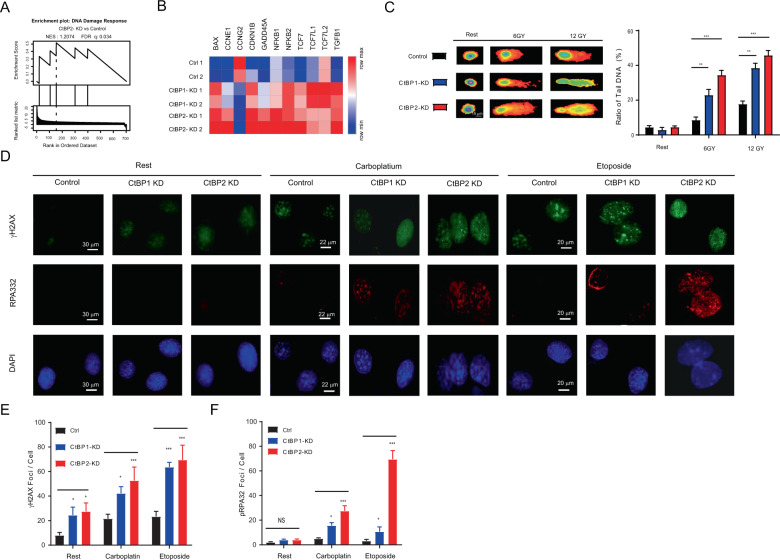
CtBP1/2 knockdown increased the sensitivity to DNA damage response in ovarian cancer cells. The upregulated genes in CtBP2 KD vs. control were enriched in DNA damage response as assessed by GSEA (**A**). Heat maps significantly compared expression of genes that involved in DNA damage response regulation in CtBP1/2 KD and control groups (**B**). Comet assay analyzed the DNA damage response the after g-irradiation treatment (6 and 12 Gy) on ovarian cancer cells. The ratio of tail DNA was as quantitative analysis (**C**). Immunofluorescence of γH2AX and RPA32 phosphorylation detected in control and CtBP1/2 KD cells after cell treated with carboplatin and etoposide (**D**). Nuclear DNA was counterstained with DAPI. Quantitative analysis foci formation per cell in each group was performed by Image J and presented as γH2AX foci/cell (**E**) and RPA32 foci/cell (**F**). Rest represented without stimulation. For each sample, 100 cells were blinded and scored independently by two markers. *P < 0.05; **P < 0.01; ***P < 0.001.

DDR-signaling pathway was significantly positive enriched (NES:1.2074, FDR q:0.034) pathways in the CtBP2-KD cell (Fig. 3A). 11 key genes involved in DDR were significantly changed, with ten genes upregulated (BAX, CCNE1, CDKN1B, GAPP45A, NFKB1, NFKB2, TCF7, TCF7L1, and TGFB1) and one gene downregulated (CCNG2). CtBP1-KD cells showed a similar change pattern without significant enrichment (Fig. 3B). Key regulatory genes, NFKB1, NFKB2, TCF7, and TCF7L1, were significantly upregulated, which are involved the DDR and oxidative stress through WNT/beta-catenin signaling pathway [23, 24]. There was no significant difference in the ratios of tail DNA between CtBP1/2-KD and control cells. The ratio of tail DNA was significantly increased in CtBP1-KD and CtBP2-KD cells after γ-irradiation (6 and 12 Gy) treatment (Fig. 3C). CtBP2-KD cells were more sensitive to γ-irradiation and generated the highest ratio of tail DNA (6 Gy, 35.8%, *P* < 0.001; 12 Gy, 45.8%, *P* < 0.001).

The γH2AX and RPA32 phosphorylation foci signals were employed as readout indicators to evaluate the impact of CtBP1/2 knockdown on the DNA damage and genomic stability in cancer cells, which were typical markers for DNA damage [25], genomic stability, and cell survival [26]. Under rest conditions (without any treatment), the γH2AX phosphorylation foci number per cell was significantly increased in CtBP1-KD and CtBP2-KD cells (Fig. 3D, E). The RPA32 phosphorylation foci signal was almost negative in these cells, with no statistically significant difference (Fig. 3F). The intensity of γH2AX and RPA32 phosphorylation foci signal became brighter after treated with carboplatin or etoposide (Fig. 3D) and the number of foci per cell increased significantly, especially in CtBP2-KD cells (Fig. 3E, F). CtBP1/2 knockdown was thought to increase genetic instability and activate DNA-dependent protein kinase (DNA-PK) in the DDR induced by carboplatin or etoposide treatment.

### CtBP1/2 knockdown shortened the DNA replication fork and increased instability

**Fig. 4:**
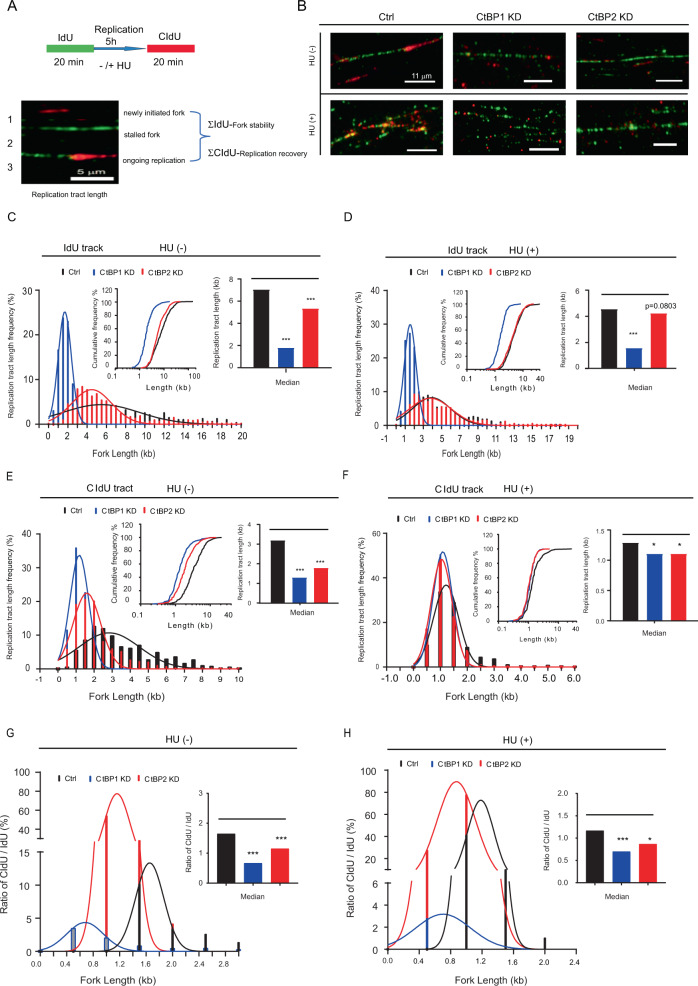
CtBP1/2 KD significantly decreased the length of nascent DNA strands at stalled replication forks. Schematic of single DNA fiber analysis experimental design. Green tracts, IdU; red tracts, CldU. Examples of various types of tracts are shown (**A**). The retention of IdU label was measured, with or without HU treatment, and reflected the stability of stalled forks (Σ IdU). The lengths of CldU tract formed after breakage have been measured (ΣCIdU), with or without HU exposure, to evaluate the impact of CtBP1/2 knockdown on the DNA replication recover ability in serous ovarian cancer cells. Demonstration of representative images of ldU/CldU tract, with or without HU treatment, in the control, CtBP1 KD, and CtBP1 KD groups (**B**). IdU tract length distributions from DNA fibers from control and CtBP1/2 KD cells in the presence (replication stalling) (**C**) or absence (unperturbed replication) of HU (**D**). CIdU tract length distributions from DNA fibers from control and CtBP1/2 KD cells in the presence (replication stalling) (**E**) or absence (unperturbed replication) of HU (**F**). Distribution curves of the ratio of CldU/IdU tract lengths with (**G**) or without HU in cells (**H**). Median tract lengths and cumulative distributions were given in parentheses here and in subsequent figures. Error bars represented the standard error of the mean (SEM).

The DNA fiber assay was used to evaluate the impact of CtBPs knockdown on genomic stability. The retention of IdU label was measured (Σ IdU), with or without HU treatment, as described in the “Methods” section, to reflect the stability of stalled forks (Fig. 4A). Without HU treatment, the median IdU tract length of CtBP1-KD/CtBP2-KD cells had significantly shorter (1.7793 kb, CtBP1-KD; 5.3212 kb, CtBP2-KD, respectively, *P* < 0.0001, two-tailed Mann–Whitney *U* test) than control cells (7.0396 kb) (Fig. 4B, C, Table 1A). HU treatment resulted in a significantly shorter median IdU tract length (1.5592 kb, CtBP1-KD) than the control (4.5338 kb, *P* < 0.0001, two-tailed Mann–Whitney *U* test), despite the fact that (Fig. 4B, D, Table 1B), although HU treatment resulted in a significantly shorter median IdU tract length in the CtBP1-KD/CtBP2-KD and control cells (Table S2). It is proposed that CtBP1/2 contribute to maintain the integrity of DNA replication track, while CtBP1 play an important role in the protection of stalled forks integrity under metabolic stress condition during prolonged periods of replication.

**Table 1:** The histogram comparison of IdU/CIdU labeled track among different groups.

Group	Ctrl	CtBP1 KD	CtBP2 KD
(A)			
Fiber labeled	IdU	IdU	IdU
Hu treatment	No	No	No
Total number of values	463	486	564
Number of excluded values	0	0	0
Number of binned values	463	486	564
Minimum	1.4789	0.4921	1.1241
25% Percentile	3.9083	1.3416	3.5703
Median	7.0396	1.7793	5.3212
75% Percentile	12.4812	2.6548	8.5748
Maximum	152.3000	17.0759	46.5034
Mean	10.1338	2.3528	7.1760
Std. deviation	10.7540	1.9222	5.6054
Std. error	0.4998	0.0872	0.2360
Lower 95% CI of mean	9.1516	2.1815	6.7123
Upper 95% CI of mean	11.1159	2.5241	7.6396
*P* value (compared with control)		<0.0001	<0.0001
Exact or approximate *P* value?		Gaussian approximation	Gaussian approximation
*P* value summary		***	***
Are medians signif. different? (*P* < 0.05)		Yes	Yes
One- or two-tailed *P* value?		Two-tailed	Two-tailed
Sum of ranks in columns		314700, 136100	261572, 266306
Mann–Whitney *U* test		17760	107000
(B)			
Fiber labeled	IdU	IdU	IdU
Hu treatment	HU	HU	HU
Total number of values	368	467	323
Number of excluded values	0	0	0
Number of binned values	368	467	323
Minimum	0.8832	0.4403	0.4921
25% Percentile	2.9681	1.1888	2.6470
Median	4.5338	1.5592	4.2139
75% Percentile	7.2494	2.1730	6.6304
Maximum	58.7360	8.4071	24.1647
Mean	5.9196	1.7409	5.2163
Std. deviation	5.2803	0.9031	3.5468
Std. error	0.2753	0.0418	0.1973
Lower 95% CI of mean	5.3783	1.6588	4.8281
Upper 95% CI of mean	6.4609	1.8230	5.6046
P value (compared with control)		*P* < 0.0001	*P* = 0.083
Exact or approximate *P* value?		Gaussian approximation	Gaussian approximation
*P* value summary		***	ns
Are medians signif. different? (*P* < 0.05)		Yes	No
One- or two-tailed *P* value?		Two-tailed	Two-tailed
Sum of ranks in columns		227800, 121300	131900, 107200
Mann–Whitney *U* test		11990	54890
(C)			
Fiber labeled	CIDU	CIDU	CIDU
Hu treatment	No	No	No
Total number of values	464	550	607
Number of excluded values	0	0	0
Number of binned values	464	550	607
Minimum	0.3522	0.2202	0.3108
25% Percentile	2.0927	0.9350	1.2484
Median	3.1818	1.2872	1.7793
75% Percentile	5.2940	1.8726	2.7273
Maximum	21.2328	12.8050	14.7241
Mean	4.0334	1.6641	2.2644
Std. deviation	2.8261	1.3866	1.6607
Std. error	0.1312	0.0591	0.0674
Lower 95% CI of mean	3.7756	1.5480	2.1320
Upper 95% CI of mean	4.2912	1.7803	2.3968
*P* value (compared with control)		*P* < 0.0001	*P* < 0.0001
Exact or approximate *P* value?		Gaussian approximation	Gaussian approximation
*P* value summary		***	***
Are medians signif. different? (*P* < 0.05)		Yes	Yes
One- or two-tailed *P* value?		Two-tailed	Two-tailed
Sum of ranks in columns		324900, 189700	316700, 257300
Mann–Whitney *U* test		38170	72790
(D)			
Fiber labeled	CIDU	CIDU	CIDU
Hu treatment	HU	HU	HU
Total number of values	389	466	382
Number of excluded values	0	0	0
Number of binned values	389	466	382
Minimum	0.4403	0.2202	0.3108
25% Percentile	0.9868	0.8832	0.8832
Median	1.2872	1.1033	1.1033
75% Percentile	1.7793	1.4116	1.3416
Maximum	27.7933	3.1572	5.0790
Mean	1.7474	1.1733	1.1504
Std. Deviation	1.9343	0.4441	0.5018
Std. Error	0.0981	0.0206	0.0257
Lower 95% CI of mean	1.5546	1.1329	1.0999
Upper 95% CI of mean	1.9402	1.2138	1.2009
*P* value (compared with control)		*P* < 0.0001	*P* < 0.0001
Exact or approximate *P* value?		Gaussian approximation	Gaussian approximation
*P* value summary		***	***
Are medians signif. different? (*P* < 0.05)		Yes	Yes
One- or two-tailed *P* value?		Two-tailed	Two-tailed
Sum of ranks in columns		190448, 175492	172800, 124800
Mann–Whitney *U* test		66680	51610

Simultaneously, the lengths of CldU tract formed after breakage were measured, with or without HU exposure, to evaluate the impact of CtBP1/2 knockdown on the DNA replication recover ability in serous ovarian cancer cells. Without HU exposure, CtBP1/2 knockdown significantly decreased the median CIdU tract length (3.1818 kb, control) (CtBP1-KD, 1.2872 kb, *P* < 0.0001; CtBP2-KD, 1.7793 kb, *P* < 0.0001, two-tailed Mann–Whitney *U* test) (Fig. 4E, Table 1C). CtBP1/2 knockdown was strongly suggested to increase the instability of DNA replication recovery and shorten the CldU tract lengths. Similar to the IdU response pattern, the replication fork recovery rate was significantly slowered with HU treatment, and the length of CldU track was significantly decreased (Table S3). The HU-induced stress of deoxyribonucleoside triphosphate pool did not alter the significantly different pattern of CldU tracks between the CtBP1-KD/CtBP2-KD and control cells (Fig. 4F, Table 1D).

Without HU treatment, the CldU tracts length were longer that the IdU track’s in control cells (median CldU/IdU = 1.725), whereas the CldU/IdU ratio was lower than 1.0 in CtBP1-KD cells (median CldU/IdU = 0.6774, *P* < 0.0001) and greater than 1.0 in CtBP2-KD cells (median CldU/IdU = 1.161, *P* < 0.0001, Fig. 4G). Although HU treatment reduced the CldU/IdU ratio of CtBP1/2 KD cells to less than 1.0 (CtBP1 KD, median = 0.7052, *P* < 0.0001; CtBP2 KD, median = 0.8738, *P* < 0.05; control, median = 1.174), it had no effect on the difference pattern between the CtBP1/2 KD and control cells (Fig. 4H). It was implied that CtBP1/2 protect against the degradation of stalled replication forks with opposite directions for the leading and lagging strands and CtBP2 appears to play a dominant protective role in serous ovarian cancer cells.

### CtBP1/2 knockdown synergistically activated DNA-PK in DNA damage response

DNA-PK activity was necessary for the NHEJ pathway [27]. Two DNA-PK selective inhibitors, KU-0060648 and NU7441, were employed as probe to investigate the regulatory role of DNA-PK during DDR in the CtBP1/2 KD ovarian cancer cells. KU-0060648 was a dual inhibitor of DNA-PK and PI3K that could increase the sensitivity of cancer cells to DNA damage induced by cytotoxic drug [27], such as etoposide. NU7441 was an ATP-selective competitive inhibitor of DNA-PK and had no inhibitory effect on the DNA-PK-related enzymes ATM and ATR even at 100 μM concentration [28]. To evaluate the contribution of DNA-PK in CtBP1/2 KD induced DDR, cytotoxic assay and fluorescence foci assay were employed to quickly explore the optimal dosage of specific or non-specific DNA-PK inhibitors for CtBP1/2 KD cells.

**Fig. 5:**
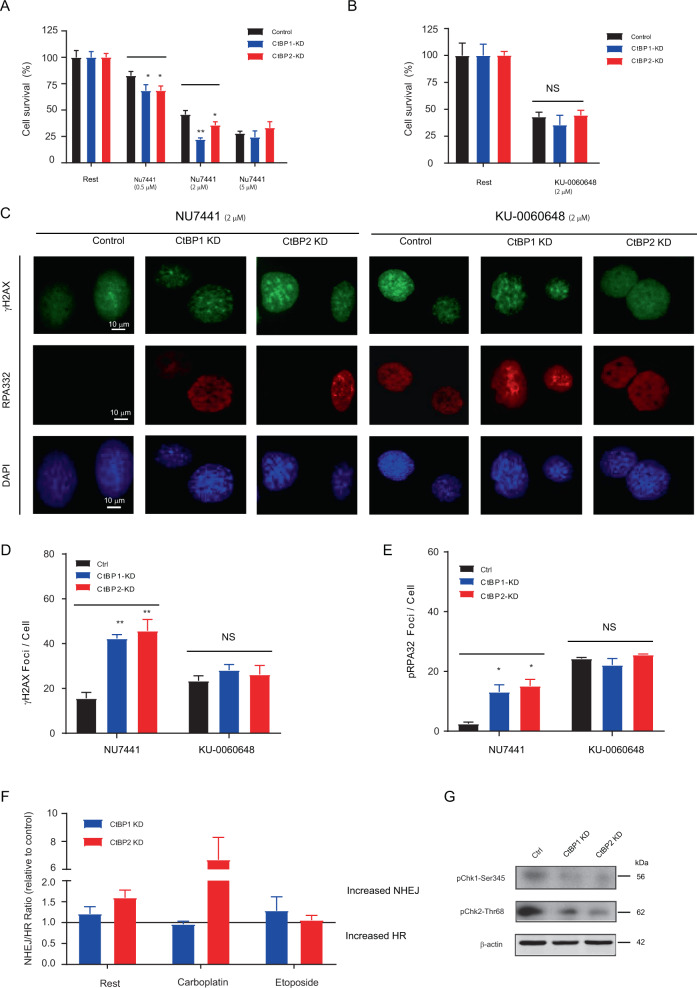
CtBP1/2 KD significantly increased DNA repair response to chemotherapy drug. The cell survival comparison between CtBP1/2 KD and control the response to DNA-PK inhibitor NU7441 (**A**) and KU treatment (**B**). Immunofluorescence of gH2AX and RPA32 phosphorylation detected in control and CtBP1/2 KD cells after cell treated with ENA-PK inhibitor NU7441 and KU000487. Nuclear DNA was counterstained with DAPI (**C**). Quantitative analysis foci formation per cell in each group was performed by Image J and presented as γH2AX foci/cell (**D**) and RPA32 foci/cell (**E**). *P < 0.05; **P < 0.001. The contribution of CtBP1/2 in the regulation of balance shifted between homology-dependent vs. homology-independent repair model in ovarian cancer cells (**F**). The SeeSaw 2.0 reporter was employed to detect the balance shift between homology-dependent vs. homology-independent repair among CtBP1/2 knockdown and control cells in rest condition, treated with carboplatin and etoposide. To measure the deviation from the balance between NHEJ and HR, the ratio between green vs. red cells in each condition was calculated. To facilitate comparing experiments, this ratio was normalized shRNA control. Those skewed the balance towards an increase in homology-independent repair has a fold-increase of over 1, while those with an increase in HR have a fold-decrease of less than one. Data represent a minimum of three sets of duplicated experiments. Western blot analyzed the key regulators between CtBP1/2 knockdown and control (**G**). Rest represents without stimulation.

NU7441 treatment significantly decreased survival and differentiated the cytotoxic response among CtBP1-KD (0.5 μM, 68.3 ± 5.80%, *P* < 0.05; 2 μM, 22.1 ± 1.48%, *P* < 0.001; 5.0 μM, 24.3 ± 5.97%, N.S), CtBP2-KD cells (0.5 μM, 68.4 ± 4.50%, *P* < 0.05; 2 μM, 35.6 ± 3.30%, *P* < 0.05; 5.0 μM, 32.1 ± 5.89%, N.S) and control cells (0.5 μM, 82.5 ± 4.08%; 2 μM, 45.8 ± 3.87%; 5.0 μM, 27.9 ± 3.70%), respectively (Fig. 5A). There was no significant difference in cytotoxic response between the CtBPs knockdown and control groups when treated with a higher dosage (5 μM) of NU7441. The dosage (2 μM) of NU7441 was chosen for foci assay. KU-0060648 exhibits differential growth inhibitory effects but not profoundly cytotoxic in multiple human cancer cell lines, and it works as a dual DNA-PK (IC50 = 8.6 nM) and PI3Ks inhibitor (IC50 = 0.59 μM for PI3Kγ) [27]. Although KU-0060648 (2 μM) treatment induced cytotoxicity in CtBP1-KD (35.5 ± 9.00%, *P* = 0.4934), CtBP2-KD (44.4 ± 4.80%, *P* = 0.8374) and control cells (43.0 ± 4.40%), there was no significant difference between different groups (Fig. 5B). The dosage (2.0 μM) of KU-0060648 was intended to completely block the DNA-PK and PI3Ks contribution in CtBP1/2-KD cells and is employed to evaluate the DDR via the foci assay.

Nuclear γH2AX and RPA32 phosphorylation foci number/cell were calculated and compared between CtBP1-KD/CtBP2-KD and control cells after 24-h treatment KU-0060648/NU7441 (2 μM) (Fig. 5C). The γH2AX foci number/cell significantly increased in CtBP1/2-KD cells (*P* < 0.001, Fig. 5D). KU-0060648 treatment increased the γH2AX and RPΑ32 foci number slightly but not significantly (Fig. 5C–E). NU7441 significantly increased the number of γH2AX and RPΑ32 phosphorylation foci in CtBP1/2-KD cells than control cells (Fig. 5C–E). KU-0060648 had the potential to increase the rate of homology-directed repair while inhibiting the NHEJ repair shift [27]. In light of these findings, it is proposed that DNA-PK be activated for the initial DNA repair and significantly increase the sensitivity to the specific inhibitor of DNA-PK in the CtBP1/2 knockdown serous ovarian cancer cells.

### CtBP1/2 knockdown regulated NHEJ/HR pathway shift in DSBs repair

To explore the impact of CtBPs knockdown in DSBs repair, the SSR 2.0 reporter was used to evaluate the NHEJ/HR pathway shift in shRNA-mediated CtBP1/2 KD serous ovarian cancer cells, which is a genetic encoding sensor that specifically measures the shift of NHEJ or HR pathway by I-SceI-induced DSBs repair [29] (Fig. S3A). Under rest condition, the NHEJ/HR ratio in CtBP1/2 KD cells was identified to be greater than 1.0 when normalized with scrambled shRNA control (1.21 ± 0.17, CtBP1-KD; 1.59 ± 0.18, CtBP2-KD) (Fig. S3B). As a result, CtBP1/2 KD shifted the balance to NHEJ pathway (Fig. 5F). Carboplatin induces high-cytotoxicity DNA lesions in cells, which is mainly repaired by HR pathway [30]. Carboplatin or etoposide treatment induced a different repair pathway balance shift in CtBP1/2 KD cells. CtBP2-KD cells dramatically shifted to NHEJ pathway (NHEJ/HR ratio = 6.69 ± 1.69) and CtBP1-KD slightly inverted to HR pathway (NHEJ/HR ratio = 0.96 ± 0.07) after carboplatin (200 μM, 24 h) treatment (Fig. S3C). Etoposide bound to topoisomerase II (TOP2) and induces DSBs, which were primarily repaired by NHEJ pathway, while BRCA1 promoted the removal of TOP2-cleavage complexes from DSBs [31]. Treated with etoposide (50 μM, 24 h), CtBP1-KD cells shifted slightly from HR to NHEJ pathway (NHEJ/HR ratio = 1.28 ± 0.33), whereas CtBP2-KD cells remained in the NHEJ pathway (NHEJ/HR ratio = 1.058 ± 0.11) (Fig. S3D, Fig. 5F).

Furthermore, western blot analysis revealed that the phosphorylation levels of Chk1-Ser345 and Chk2-Thr68 were significant lower in CtBP1/2 KD cells than the control cells (Fig. 5G). It was hypothesized that the activity of Chk1 and Chk2 would be suppressed in the CtBP1/2 KD cells. As a key molecule of DDR-signaling downstream and DNA damage marker, phosphorylation level of Chk1-Ser345 was crucial for Chk1 activation and necessary for HR repair [32]. When exposed to ionizing radiation or UV, phosphorylate level of Chk2-Thr68 increased, causing the pathway switch from the NHEJ to error-free HR. DNA-PK controlled the Chk2-Thr68 phosphorylation level and regulated the Chk2–BRCA1 pathway to reverse pathway shift and ensured chromosomal stability [33]. We proposed that CtBP2 would play a dominant role in maintaining genetics stability and regulate NHEJ/HR pathway shift in DSBs repair.

### Patients with CtBP2 genetic alterations significantly extended the overall survival time

To validate the hypothesis that disrupting CtBP2’s function may inhibit the abnormal growth of cancer cells, we utilized the TCGA via cBioportal to explore the potential correlation of CtBP1/2 genetic alterations with patient’s overall survival time in serous ovarian cancer patients. There are total of 153 (~10.0%, Table S4) patients associated with CtBP1/2 genes genetic alternation among 1680 cases, 78 (5%) in CtBP1 and 75 (5%) in CtBP2, respectively. Interesting, patients with CtBP2’s genetic alternation were significantly associated with longer overall survival time (median time: 61.67 months, log rank test, *P* value: 3.198e−4, *n* = 72) than unaltered patients (median time: 44.48 months, *n* = 1561) in ovarian serous cystadenocarcinoma patients during 10-year survival period (Fig. S4B). While CtBP1-related alterations did not significantly impact the overall survival time (median time: 52.0 months, log rank test, *P* value: 0.358, *q* value: 0.613, *n* = 78) than unaltered patients (median time: 44.55 months, *n* = 1555, Fig. S4A). These genetic alternations of CtBP1/2 were summarized, including P308H missense mutation and LYAR fusion in CtBP1, and K434Nfs*33 frameshift deletion and KCNMA1-CtBP2 gained fusion merged in CtBP2 (Table S4), including fusion, amplification and homo deletion, missense mutation, and copy-number alterations, which are primarily distributed in ovarian cancer and ovarian epithelial cancer. Recently, genomics features of several ovarian cancer cell lines were well characterized and identified typical genetic alternations in these cancer cells, which are broadly used in research, including SKOV3 and OVSAHO cells. More interesting, the typical genes deletions of NOTCH1, CDKN2A and TP53 in SKOV3 cells [34] were significantly associated with CtBP2’s alternation in the CtBP2 altered patients’ profile (Table S5). It was more convincible and clinical relevant that CtBP2’s genetic alternation would disrupt its function and attenuate it abnormal impact in serous ovarian cancer.

## Discussion

In this study, multiple research techniques, including shRNA-based stable CtBP1/2 knockdown, transcription profiles analysis and DNA Fiber, et al., were applied to investigate the contribution of CtBP1/2 in apoptosis, genomic stability, and DDR of serous ovarian cancer cells. Initially, the study purpose was to investigate the contribution of the CtBP proteins play in the HGSOC. According to previously findings, overexpression of CtBP2 was linked with abnormal proliferation, epigenetically silencing BRCA1 function [21], and poorer survival rate in ovarian cancer patients [13]. CtBP1/2 were found to be overexpressed in multiple subtypes of ovarian cancer cells by western blot analysis (Fig. 1A). The SKOV3 cell was chosen as model to investigate the role of CtBP1/2 in HGSOC by integrated shRNA-based stable knockdown and transcription profiles analysis (Fig. 1B, C). The most significantly enriched functional pathways in CtBP1/2 KD cells were mainly concentrated on cell cycle regulation, adhesion, DDR, and apoptosis, according to enrichment analysis for significant change genes (Fig. 1D–E). The abilities of proliferation (Fig. S2A), cell migration and clone-forming in vitro (Fig. S2B–E) were significantly reduced in stable CtBP1/2 KD cells. Single CtBP1-KD or CtBP2-KD induced a high portion of apoptosis (Fig. 2C, D), whereas CtBP1/2 DKD accelerated the apoptosis. CtBP proteins had been identified as anti-apoptotic proteins and negatively regulator through several program cell death genes, such as caspase-3, BIK and D3R, in human cancer cells [17, 35, 36]. In our results, TGF-b was found to be significantly upregulated in the CtBP1/2 KD cells (Figs. 2B and 3B). CtBP1/2 KD increased the metabolic stress and DNA replication instability, and could activate HIPK2, a serine /threonine kinase in the TGF-β signaling pathway, by checkpoint kinase ATM under stress and leading to apoptosis in cancer cells, which directly or indirectly (via JNK1) phosphorylates the sites of Ser422 and Ser428 in CtBP1 and CtBP2 [36–38], respectively. It may create a feedback loop, exaggerating and hastening the apoptosis in CtBP DKD cells. CtBP proteins were thought to act as apoptosis suppressers and interact with TGF-β signaling pathway in serous ovarian cancer cells. It could be partially explained that genetic alterations in CtBP2 producing abnormally functional protein and inducing tumor apoptosis, thereby extending the patients’ survival time.

CtBP proteins enhanced mitotic fidelity and genome stability through their metabolic activity in the nucleus [14]. However, it is unclear what role CtBP1/2 in DNA replication and DNA damage repair in serous ovarian cancer cells. In this study, DDR-signaling pathway was highlighted and positively enriched in CtBP2-KD cells rather than in CtBP1-KD cells (Fig. 1D, E). CtBP1/2 knockdown significantly increased the genomic instability and the sensitivity to γ-irradiation, especially in CtBP2 KD cells (Fig. 3C). It could be attributed to difference in intracellular distribution and metabolic protective functions of CtBP1/2. CtBP2 played dominant localized in nuclear and works as transcriptional corepressors [39, 40]. Furthermore, CtBP1/2 knockdown significantly disrupted the stability of the DNA replication fork and increased the instability of DNA replication recovery in serous ovarian cancer cells (Fig. 4C–F). Under the metabolic stress condition (HU treatment), CtBP1 had a greater protective effect than CtBP2’s (Fig. 4D, F).

Approximately 44% of HGSOC patients had HR deficiency (24). Defects in DNA repair were likely to affect the sensitivity of platinum in HGSOC [4]. DSBs were most of the cytotoxicity response to genotoxic stress. NHEJ and HR pathways mainly involved in the DNA repair of DSBs. NHEJ pathway was a highly error-prone DNA repair mechanism that operates throughout the cell cycle [41]. DNA-PK was a mediator of NHEJ and specific DNA-PK inhibitors were widely used in platinum-resistant cancer cell lines as a chemo-sensitization strategy [42]. In this study, CtBP1/2 knockdown increased genetic instability and activated DNA-PK during DNA breakage. The preference of DNA repair pathway shifted from HR to NHEJ under resting condition. Chemotherapy drugs (Carboplatin and Etoposide) treatment had an effect on the trend of NHEJ pathway shift (Fig. 5F, Fig. S3B–D). CtBP2 knockdown did not enhance the carboplatin cytotoxic effect in serous cancer cells (data not shown). A synergic effect of a specific DNA-PK inhibitor and CtBP2 KD would exist in serous ovarian cancer cells. Based on these findings, a novel potential treatment strategy, the combination of specific CtBP2 protein inhibitor and DNA-PK inhibitors, was proposed to target CtBP2 protein and DNA-PK in platinum-resistant serous ovarian cancer.

We investigated the correlation of CtBP1/2 genetic alterations with patient overall survival time in serous ovarian cancer patients using TCGA cases to validate the results that disruption of CtBP proteins function would lead to cancer cell apoptosis and increase DNA instability. Although CtBP1 and CtBP2 had similar levels of genomic alterations (around 5%), CtBP2’s genetically altered cases had significantly longer survival time (Fig. S4B) than unaltered cases. Previously, CtBP2 overexpression was linked with poorer survival rate in invasive ovarian cancer [13]. Oncogene amplification and overexpression are not the same thing. The amplification copy of a gene may be translocated into parental alleles or other chromosome(s) or extra-chromosome acentric elements [43]. Gene overexpression could be caused by a variety of epigenetic and genetic modifications [44, 45], including copy number increase and paternal gene overactivation, etc. Oncogene’s overexpression may not be associated with detectable amplification. For example, the oncogene MET was amplified with 5.1% cases while overexpressed in 13.1% cases of glioma [46]. Gained-fused KCNMA1-CtBP2 gene may destroy the function of KCNMA1, which was overexpressed in ovarian cancer cells and promoted proliferation, migration and attenuation of apoptosis [47], and induced apoptosis in ovarian cancer cells. It is possible that genetic alterations of CtBP2 destroy the abnormal activity of CtBP2 in serous ovarian cancer, resulting in a longer survival time for patients with the disease. More intriguing, some typical genetic alternations of SKOV3 cell [34], such as deletion of NOTCH1, CDKN2A, and TP53 genes, were significantly associated with CtBP2’s alternation in the CtBP2 altered patients’ group (Table S5). It was more convincible and clinical relevant that CtBP2’s genetic alternation would disrupt its function and attenuate it abnormal impact in serous ovarian cancer. Up to now, little is known about the exactly mechanism linking between CtBP2’s genetic alternation and significantly longer overall survival time. It will be an exciting new research direction for CtBP2 role in ovarian cancer, with greater potential translational application for ovarian cancer patients.

In summary, this study discovered that CtBP1/2 played differentially protector of genetic stability and DNA repair pathways in serous ovarian cancer cells. CtBP1/2 contributed to maintain the stability of DNA replication fork and DNA repair in serous ovarian cancer cells. CtBP2-related genetic alterations may destroy its abnormal activity and abolish the proliferation-promoting effect in serous ovarian cancer. Combined with these results, it will give a new perspective on the regulatory role of CtBP proteins in genetic stability and DNA repair pathway in ovarian cancer. Advance knowledge of ovarian cancer deregulatory mechanism will accelerate the translational application and development of novel promising clinical treatment strategy and solutions for serous ovarian cancer patients.

## Materials and methods

### Ovarian cancer cells culture

Human ovarian cancer cell lines (HOSE, SKOV3, MCAS, RMG1, and RMUGL) were purchased from ATCC and cultured in DMEM medium or RPMI 1640 supplemented with 10% fetal bovine serum, 0.1 mg/mL penicillin, and 0.1 mg/mL streptomycin.

### Statistical analysis

Data were presented as means ± SEM. Significance of differences for the values was determined using the Student’s *t*-test with Prism software (GraphPad Software, Inc. San Diego, CA). A *P* value less than 0.05 was considered a significant difference.

Detailed descriptions of the following techniques were available in the Supplementary Materials and Methods:

1. Established CtBP1/2 stable knockdown ovarian cancer cells
2. Western blotting
3. Whole transcript expression profiling analysis
4. Proliferation, drug treatment, wound scratch, and transwell assay
5. Colony-forming assays
6. Cell cycle analysis
7. DNA fiber assay
8. Immunofluorescence image analysis for DNA damage response
9. Comet assay for DNA Damage induced by Irradiation
10. NHEJ and HR repair pathways shift Assay
11. Explore CtBP1/2 gene with serous ovarian carcinoma cases through TCGA

## Supplementary information

Supplementary materials and methods

Supplementary Figure X Table legend

Figure S1

Figure S2

Figure S3

Figure S4

Table S1

Table S2

Table S3

Table S4

Table S5
