# Supplementary material for: CtBP1/2 differentially regulate genomic stability and DNA repair pathway in high-grade serous ovarian cancer cell
Source: Oncogenesis. 2021 Jul 13;10(7):49. doi: 10.1038/s41389-021-00344-9 (PMC8275597; doi:10.1038/s41389-021-00344-9)
Supplement: Supplementary file 1 — Supplementary materials and methods [file 41389_2021_344_MOESM1_ESM.pdf]

CtBP1/2 differentially regulate genomic stability and DNA repair pathway in high-grade serous ovarian cancer cell

Yingying He[2], Zhicheng He[1,3], Jian Lin[1,3], Cheng Chen[1,3], Yuanzhi Chen[1,3], Shubai Liu[1,3,#]

[1.] State Key Laboratory of Phytochemistry and Plant Resources in West China, Kunming Institute of Botany, Chinese Academy of Sciences, Kunming, 650201 Yunnan, P. R. China.

[2.] School of Chemical Science & Technology, Yunnan University, Kunming, Yunnan 650091, China.

[3] University of Chinese Academy of Sciences, Beijing 100049, China.

Supplementary Materials and Methods:

Established CtBP1/2 stable knockdown ovarian Cancer Cells

The stable knockdown CtBP1 or CtBP2 ovarian cancer cells (1 x 10^5 cell / well, SKOV3) were established by infected with lentiviral CtBP1/2-targeting shRNA and non-target control shRNA transduction particles 72 hours (Sigma-Aldrich, St. Louis, MO) and screened with 2 μg/ml puromycin for 2 weeks. The survival cells were maintained in a DMEM medium (Sigma, St. Louis, MO) supplemented with 10% fetal calf serum and 2 μg/ml puromycin (Invitrogen, Carlsbad, CA). In order to generate a dual knockdown of the CtBP 1/2 ovarian cancer cell, the CtBP1/2 shRNA swap was applied to the positive knockdown clones and failed to generate double knockdown clones. The transient double knockdown CtBP1/2 was achieved by transfected with siRNA targeting CtBP1 (GGAUAGAGACCACGCCAGUUU, Sangon Biotech) and CtBP2 (CCUUUGGAUUCAGCGUCAUTT, Sangon Biotech) in stable CtBP1/2 alternative knockdown cancer cells with 72 hours and harvested cell for next step test. The expression levels of CtBP1/2 were tested by Western blot analysis.

Western blot analysis

Cells were washed twice with ice cold PBS and lysed on ice with lysis buffer supplemented plus protease inhibitor cocktail (Cell Signaling, Danvers, MA). Samples containing 40 μg of total protein (BCA, Pierce, Rockford, IL) were separated on SDS/PAGE (Invitrogen, Grand Island, NY) and transferred to a PVDF membrane (Bio-Rad, Hercules, CA). Target proteins were visualized by primary antibody binding

[CtBP1, CtBP2, pCDC2-Y15, CDK2, pChk1-Ser345, pChk2-Thr68] (Cell signaling Technology, Danvers, MA) followed by secondary antibody binding (IRDye 680CW goat-anti mouse or IRDye 800CW goat-anti rabbit) on an Odyssey scanner (LI-COR biosciences, Lincoln, NE). The beta-actin (Abcam, Cambridge, MA) was used as internal loading control bands, and each result represents multiple time repeat.

Whole transcript expression profiling analysis

RNA was extracted from SKOV3 shRNA control and CtBP1/2 knockdown cell lines using TRIzol reagent (Invitrogen, Carlsbad, CA). The quality and quantity of the RNA were tested using spectrophotometric analysis and Bioanalyzer (Agilent Technologies, Santa Clara, CA). RNA was extracted from cell lines using TRIzol reagent (Invitrogen, Carlsbad, CA). 1 ug of RNA per each sample were used for target labeling by a two-round amplification protocol. Expression profiles were determined using Affymetrix 1.1 Human Gene ST arrays according to the manufacturer's instructions. For RNA expression profiling, each sample toke 4.5μg of fragmented, labeled and hybridized with per GeneChip (Human Gene whole transcript 1.1 ST Arrays, Affymetrix) were processed on the Affymetrix GeneAtlas Fluidic station. The data were normalized to the median of all probe sets on each chip and lastly by “per gene”. For example, normalized to the median expression of each probe set across all samples. Expression data were normalized, background-corrected, and log2-transformed for parametric analysis. All Affymetrix control genes were removed and the remaining Affymetrix probe clusters were imported into the Affymetrix transcription analysis console.

Significantly Differentiated expression genes were identified using significance analysis of microarrays (SAM) with the R package 'samr' (false discovery rate (FDR) <0.05; fold change >2) and determining the gene list based on the number of significant genes that were identified by fold change. Two-dimensional hierarchical clusters are generated. Gene significantly enrichment analysis (GSEA) were applied to identify enrichment functions, pathway and networks.

Proliferation, drug treatment, wound scratch and transwell assay

The cell proliferation and cytotoxicity of the drugs to ovarian cancer cells were tested by tetrazolium-based MTT method (21) in time point manners. Briefly, at the beginning, the single cells solution was (5,000 cells /well) allocated into each well of 96 wells. For proliferation assay, the cells were cultured as normal, and 10μl of the MTT dye solution was added to each well after per 24h cultured to incubate at 37°C for 4 hours in a humidified chamber. For drug toxicity assay, the drugs were added into plate wells after cells were completely attached. After 48 hours of treatment, 10μl of the MTT dye solution was added to each well and the plates were incubated at 37°C for 4 hours in a humidified chamber. After incubation, 100μl of the solubilization/stop solution was added to each well. One hour after the addition of the solubilization solution, the content of the wells was mixed and read by the 96-well plate scanning spectrophotometer (μQuant) and quantitative software (KC-junior, Bio-Tek Instruments, Inc.) at an absorbance of 630 nm for quantitative analysis. The scratch wound healing was performed using a 6 well plate. The cells were cultured as for 24h to form a confluent

monolayer, then scratches were performed using a 10-μl tip and the culture medium was replaced with fresh complete medium. At the start of experiment, after 12h, 24 h and 48h of incubation, the plates were checked under microscope and took images to track the scratches width. Boyden chamber transwell assays were employed to evaluate the transmigration capability. Briefly, detached cells were washed with PBS twice and resuspended in serum-free medium for transwell assays. Plate 100 ul (1 x 10^6 cells/ml) cell solution on top of the filter membrane in 24-well transwell insert (corning) and incubate at 37°C for 10 min to allow cells to settle down. Plates were coated with substrate (Colg I) for 30 min prior to use. Assays were run for 24 h, and cells were then fixed with 70% ethanol and stained with 0.2% crystal violet. Results represents three times experiments repeats. All the images were converted to 8-bit images and analyzed using Image J programmer to quantitative calculate the scratches width.

Colony-Forming Assays

The scramble control and stable CtBP1/2 knockdown of Skov3 Cells were cultured in soft agar gel for additional 30-day cultured followed the protocol. The cancer cells formed colonies were stained with 0.5% crystal violet/20% ethanol and taken image by light microscope. The colonies numbers were counted by using Image J software.

Cell cycle analysis

For cell cycle analysis, the growth confluent cells (80%) were detached with trypsin, washed twice by PBS, and then were resuspended in ice-cold 70% ethanol and stored

at -20 °C for at least 24 h. The cells were then washed twice with 1X PBS and once with 1X PBS supplemented with 0.1% (v/v) TritonX-100 and resuspended in 50 μg ml/L PI (propidium iodide) staining buffer in the presence of 300 μg ml/L RNase A for 30 min at room temperature. Samples were analyzed using flow cytometry (Accuri C6 Biosciences).

Immunofluorescence image analysis for DNA damage response

In order to better understand the DNA damage response mechanism of CtBP protein regulated in ovarian cancer cells, two chemotherapy drugs, carboplatin and etoposide, were employed as positive control that induce DNA damage by binding with DNA (25) and interaction with nuclear topoisomerase II (26). DNA-dependent protein kinase (DNA-PK) activity is necessary for the Nonhomologous end joining (NHEJ) pathway (27). The KU-0060648 is a dual inhibitor of DNA-PK and PI3K, and could increase the sensitivity of cancer cells to DNA damage by cytotoxic drug (27), while NU7441 is an ATP-competitive inhibitor of DNA-PK and has no inhibitory effect on the DNA-PK-related enzymes ATM and ATR at a dose of 100 μM (28). Briefly, the cells were seeded in a Chamber Slides (Nalge Nunc International) and normally cultured overnight. Cells were treated with chemotherapy drug [Carboplatin and etoposide] and two DNA-PK selective inhibitors, KU-0060648 (KU) and NU7441, for 24 hours and then washed twice with PBS and changed with fresh normal medium cells incubated at 37°C for 24-hour recovery. The cells were stopped culture at different time points. Cells were washed 3 times with cold PBS and fixed with then permeabilized with 0.5% Triton X-

100 in PBS for 5 minutes at room temperature. Cells were blocked overnight at 4°C with blocking buffer (0.1% Triton X-100, 2% BSA in PBS). Blocked cells were incubated with anti-phospho-histone H2A.X (Ser139) monoclonal antibody (Millipore) and anti-RPA32 (4E4, Rabbit) for 1 hour at a 1:1,000 dilution in blocking buffer at room temperature. The γH2AX phosphorylation foci number per cell is a marker for DNA damage (29). The RPA32 phosphorylation foci signal is the downstream signaling target of DNA-PK during replication stress and acts as a marker for genome instability and cell survival (30). After washing 3 times with washing buffer (0.1% Triton X-100 in PBS), cells were then incubated for 1 hour at room temperature with (Alexa Fluor 488 goat anti-mouse, Alexa Fluor 647 goat anti-rabbit) secondary antibodies (Invitrogen) in blocking buffer. Images were visualized using Zeiss Axiovert 200 inverted fluorescence microscope (63 x oil objectives) equipped with 14-bit ECCD camera and argon and krypton gas excitation asters at 488 and 568 nm. Z-stack acquisition using optimal slice distancing was conducted on each microscope image. The images were processed and analyzed with ImageJ software (version 1.47v). Images were then compressed to produce a maximum intensity projection image to allow maximum foci detection. Foci were counted in 50 cells per time point and results are expressed as mean number of foci per cell from 3 independent experiments.

Comet assay for DNA Damage induced by Irradiation

The cells were seeded in a 3.5cm^2 dishes (Nunc International) and normally cultured overnight. IR was delivered by an X-ray generator (Faxitron X-ray Corporation RX-

650, 120 kV, 5 mA, and dose rate 6Gy, 12 Gy/min). The irradiated cells were put back to normal culture for 4 hours. Comet assay were performed according to protocol reported by Olive et al (31). Briefly, a single-cell suspension was prepared using enzyme disaggregation. Cells were washed twice with ice-cold PBS and put on ice minimize cell aggregation and inhibit DNA repair. Appropriate numbers of cells (5000 cells) were added into low-gelling-temperature agarose and a thin layer of cells will be adherent to the 16 wells chamber slides. The cells were exposed to neutral lysis buffer (2% sarkosyl, 0.5M Na2EDTA, 0.5 mg/ml proteinase K (pH 8.0); equilibrate at 4 °C) for overnight at 37°C. Wash the film with electrophoresis buffer twice and submerge the film to the electrophoresis buffer in a chamber for electrophoresis. The cells were stained by PI and the pictures were obtained using fluorescence microscopy. The tail moment was used to tell the DNA damage extent. The tail moment is calculated by the following formula: Tail moment=tail length x percentage of Tail DNA. Percentage of Tail DNA= aT×iT/(aT×iT+aH×iH), where aT represents the tail area, iT means average intensity of tail, aH represents the head area and iH means average intensity of Head. The software Comet Score^{TM} was used to analyze the comet images.

DNA Fiber Assay

To discover the role of CtBP1/2 in maintaining DNA replication fidelity and fork stability, DNA fiber analysis was used to monitor replication disturbance at single-molecule resolution. Briefly, newly synthesized DNA strands were labeled just before (green fluorescent IdU) and after (red fluorescent CldU) exposure to Hydroxyurea (HU),

which temporarily stalls DNA replication by exhausting the deoxyribonucleoside triphosphate pool (22). SKOV3 scramble control and CtBP1/2 KD Cells were split to a density of $5x10^5$ cells/ml for labeling sites of ongoing replication with IdU (25 μM) for 20 min at 37 and 5% CO_2, and washed twice with PBS, then re-suspended with normal medium, followed by exposure to hydroxyurea (Hu, 4 mM), or untreated media for up to 5 hr, as indicated in the figures. Drugs were removed and cells were washed 3 times with phosphate buffered saline (PBS) before labeling with media containing CIdU (250 μM) for 20 min to mark sites of replication recovery. The ratio of CldU/IdU between the control and CtBP1/2 knockdown cells with or without HU treatment was compared in order to discuss the degradation of replication track impact of the conduction. DNA fiber spreads were essentially performed as previously reported (23, 24) with certain modifications. Briefly, cells were harvested and resuspended in cold PBS. The cell suspension was mixed 1:6 with lysis buffer (0.5% SDS, 50 mM EDTA, 200 mM Tris-Cl) and spotted onto a microscope slide (Fisher Scientific), which was carefully tilted in a 15-degree angle to allow spreading of the genomic DNA into single molecule DNA fibers by gravity. Fibers were then fixed in methanol and acetic acid (3:1) and subsequently acid treated with HCl (2.5 N) to denature the DNA fibers. Slides were neutralized and washed with PBS (1x pH 8.0, 3x pH 7.4) before blocking with 10% goat serum and 0.1% Triton-X in PBS for at least 1 hr. Slides were incubated with primary antibodies against IdU (BD Biosciences, anti-BrdU mouse, 1:50 in blocking buffer) and CIdU (Novus Biologicals, anti-BrdU rat, 1:200 in blocking buffer) and secondary antibodies (Invitrogen, Alexa Fluor 488 goat anti-mouse, 1:200 in blocking

buffer and Alexa Fluor 647 goat anti-rat, 1:300 in blocking buffer) for 1 hr each. Slides were analyzed using an Olympus BX60 microscope (100x, oil). Fibers were analyzed using ImageJ software. The numbers of fibers were generated from independent experiments performed for each condition. The median replication tract length and p-values derived from the Mann-Whitney test were calculated using Prism software. In addition, 95% confidence intervals were calculated for each cumulative distribution, which were tested for normality using the D'Agostino-Pearson test. The rate for nascent replication tract degradation was estimated using the published conversion of 2.59 kb/mm (23). The CldU/IdU ratio was calculated and indicated the degradation of stalled replication forks. CldU tracts shorten whereas IdU tracts remain intact, CldU/IdU ratio <1.0, indicating that the more recently synthesized DNA is degraded first. In contrast, the CldU/IdU ratio > 1, means the reverse degradation.

NHEJ and HR repair pathways shift Assay

DNA double-strand breaks (DSB) are the most cytotoxic lesions caused by topoisomerase II poisons. The DSBs repair pathways are mainly processed by the balance shift between the NHEJ and HR (32). In order to discover the role of CtBP1/2 in the DSBs repair, the NHEJ and HR pathway balance shift was analyzed by SSR 2.0 reporter in shRNA-mediated CtBP1/2 knockdown ovarian cancer cells, a genetic encoding sensor that specifically measures the efficacy of NHEJ or HR repair by I-SceI-induced DSBs (33). Briefly, SSR 2.0 plasmid (form Addgene) reporters were integrated into scramble control and stable CtBP1/2 knockdown of Skov3 Cells by

plasmid transfection. Cells were cultured in high-glucose Dulbecco's Modified Eagle Medium (DMEM) supplemented with 10% FBS, 2 mM Glutamine, 100 mg/ml streptomycin, 100 U/ml penicillin, and 0.5 mg/ml G418 at 37°C in 5% CO2. After SSR 2.0 expressed 72 hours, cells were selected with 0.5 mg/ml G418 antibiotics. Positive clones were obtained and expended in 15 cm diameter plates and selection of isolated colonies. The next day, cells were infected with lentivirus particles containing I-SceI–RFP expression construct at MOI 5 using 8 mg/ml polybrene in 300 ml DMEM. After 6 hr, media was exchanged with fresh DMEM. The chemotherapy drug carboplatin (200 uM) and etopside (50 uM) treated with 24 hour and cells were washed with PBS, trypsinized, neutralized in DMEM, and centrifugated at 1000 g. Cells were resuspended, fixed with 4% paraformaldehyde for 20 min, and collected by centrifugation. Pellets were then washed twice with PBS and resuspended in 200 ml PBS. Samples were analyzed using flow cytometry (Accuri C6 Biosciences). For the HR/NHEJ balance, the ratio between green and red cells in each condition was calculated as published (22). To facilitate the comparison between experiments, this ratio was normalized with a control. Those conditions that skew the balance toward an increase in NHEJ repair result in fold change increase over 1. On the contrary, a net increase of this ratio (values below 1) represents an imbalance of the SSR toward HR (Fig.S4A). Data represent a minimum of three sets of duplicated experiments.

Explore CtBP1/2 gene with serous ovarian carcinoma cases through TCGA

The CtBP1/2 genes were explored in the Cancer Genomics dataset (TCGA) through

cBioportal and investigated the genetic alterations associated with serous ovarian carcinoma's patient's cases, which provides visualization, analysis and downloads of large-scale cancer patients genomics data sets from CCLE and TCGA to public (42, 43). Three independent cancer genomic cohort studies of serous ovarian carcinoma were found in the cBioPortal for Cancer Genomics, including Ov_tcga_pub, Ov_tcga and OV_tcga_pan_can_atlas_2018 (Table.S1), including 1680 patient serous cystadenocarcinoma datasets. In the next analysis, the selected cohort was limited to patients with serous ovarian carcinoma, and complete analysis of genomics alterations types (including single nucleotide variants, insertion/ deletion, amplification, and arrangement). The overall survival analysis comparison was performed between CtBP1/2 altered cases and unaltered related.
