# Supplementary material for: CtBP1/2 differentially regulate genomic stability and DNA repair pathway in high-grade serous ovarian cancer cell
Source: Oncogenesis. 2021 Jul 13;10(7):49. doi: 10.1038/s41389-021-00344-9 (PMC8275597; doi:10.1038/s41389-021-00344-9)
Supplement: Supplementary file 2 — Supplementary Figure X Table legend [file 41389_2021_344_MOESM2_ESM.pdf]

CtBP1/2 differentiate regulates genomic stability and DNA repair pathway in high-grade serous ovarian cancer cell

Yingying He[2], Zhicheng He[1,3], Jian Lin[1,3], Cheng Chen[1,3], Yuanzhi Chen[1,3], Shubai Liu[1,3,#]

[1.] State Key Laboratory of Phytochemistry and Plant Resources in West China, Kunming Institute of Botany, Chinese Academy of Sciences, Kunming, 650201 Yunnan, P. R. China.

[2.] School of Chemical Science & Technology, Yunnan University, Kunming, Yunnan 650091, China.

[3] University of Chinese Academy of Sciences, Beijing 100049, China.

Supplementary Figure & Table Legends:

Supplementary Figure 1. Normalization and hierarchical cluster analysis of CtBP1/2 knockdown transcription profiles in serous ovarian cancer cells. The significant changed genes were screened and identified of CtBP1KD vs control (A), CtBP2 KD vs control (B) and CtBP1/2 double KD vs control (C). each group contained the panel of filter criteria, volcano distribution of significant changed genes, and cluster analysis.

Supplementary Figure 2. CtBP1/2 knockdown impacted cell function of ovarian cancer cells.

The comparisons were performed between CtBP1/2 KD and control in SKOV3 cell proliferation (A), the clonogenicity in soft agar (B) and cell adhesion to different extracellular matrices (C), the capability of cell migration (D). These functional assays were proceeded as described in methods sections. All the data represented the average of triplicates values generated by three independent experiments. *, $P < 0.05$; **, $P < 0.001$; ***, $P < 0.0001$.

Supplementary Figure 3. Flow cytometry analysis the CtBP protein mediates DSB repair pathway shift.

Graphical Schematic representation of the SeeSaw reporter SSR 2.0 (33) (A). A GFP gene is flanked by two truncated parts of the RFP gene (RF and FP). Two I-SceI target sites were cloned at the end of the GFP gene in opposite orientation in SSR 2.0 (B).

Expression of I-SceI generates a DSB; if the damage is resolved by NHEJ, cells will express the GFP protein, while if it is repaired using homologous sequence by HR, cells express the RFP gene. Flow cytometry analysis the NHEJ/HR repair pathway shift mediated by CtBP1/2 knockdown SKOV3 cells under rest condition (without drug treatment) (B) or treated with Carboplatin (C) or Etoposide (D).

Supplementary Figure 4. Overall Survival comparison of CtBP1/2 altered patients and unaltered patients in Ovarian Serous Cystadenocarcinoma via the cBioportal Genomics database. Overall Survival comparison of CtBP1 (A) and CtBP2 (B) altered patients and unaltered patients.

Table S1. The overlapped significant changed genes among CtBP1/2 knockdown groups in SKOV3 cells.

Table S2. The Histogram comparisons of IdU labelled track between with or without Hu treatment among different groups.

Table S3. The Histogram comparisons of CIdU labelled track between with or without Hu treatment among different groups.

Table S4. The summary of CtBP1/2 genetic alterations in Serous ovarian cancer cases.

Table S5. The SKOV3's typical alternated genes enriched in the CtBP2 genetic altered cases group.
