# Supplementary material for: CtBP1/2 differentially regulate genomic stability and DNA repair pathway in high-grade serous ovarian cancer cell
Source: Oncogenesis. 2021 Jul 13;10(7):49. doi: 10.1038/s41389-021-00344-9 (PMC8275597; doi:10.1038/s41389-021-00344-9)

A

SKOV3

CTBP1 KD vs CTRL
• CT1 KD: 2 samples, CTRL: 2 samples

Filter criteria:
• Fold Change: > 1.5 or < -1.5
• P-val: < 0.05

Total number of genes: 29096
• Genes passed filter criteria: 355 (1.22%)
• Up-Regulated: 182 (51.27%)
• Down-Regulated: 173 (48.73%)

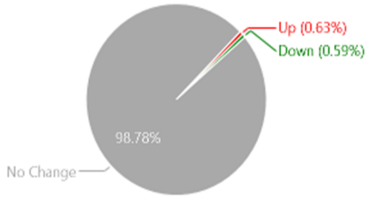

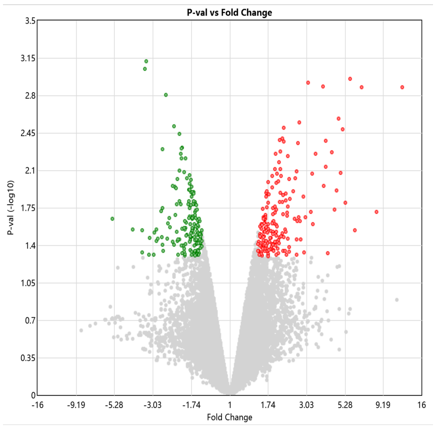

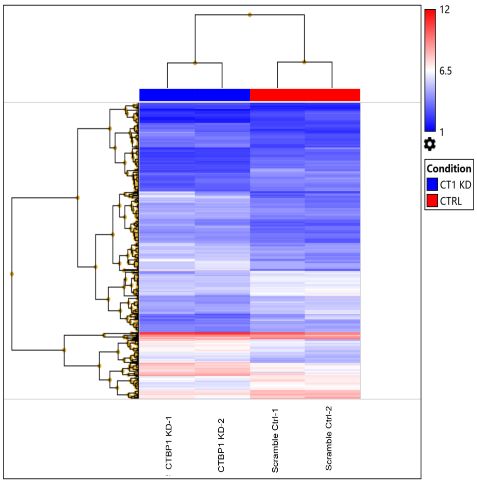

B

SKOV3

CTBP2 KD vs CTRL
• CT2 KD: 2 samples, CTRL: 2 samples

Filter criteria:
• Fold Change: > 2 or < -2
• P-val: < 0.001

Total number of genes: 29096
• Genes passed filter criteria: 805 (2.77%)
• Up-Regulated: 784 (97.39%)
• Down-Regulated: 21 (2.61%)

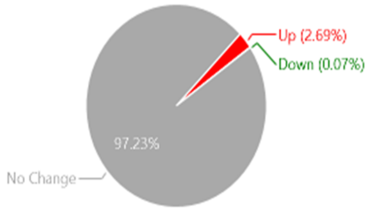

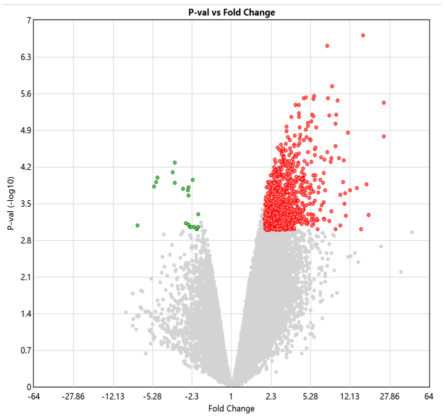

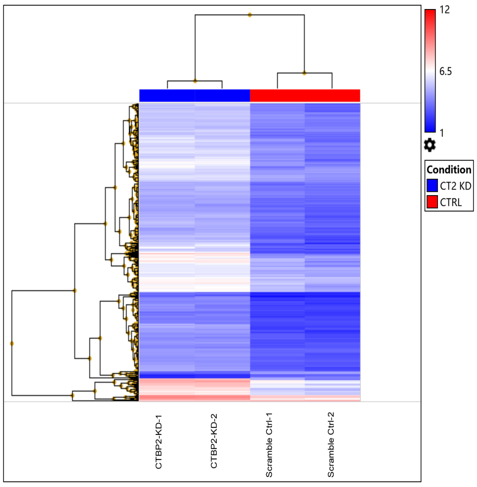

C

SKOV3

CtBP 1/2 DKD vs CTRL
• DKD: 2 samples, CTRL: 2 samples

Filter criteria:
• Fold Change: > 3 or < -3
• P-val: < 0.001

Total number of genes: 29096
• Genes passed filter criteria: 4297 (14.77%)
• Up-Regulated: 1364 (31.74%)
• Down-Regulated: 2933 (68.26%)

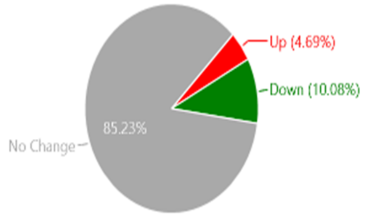

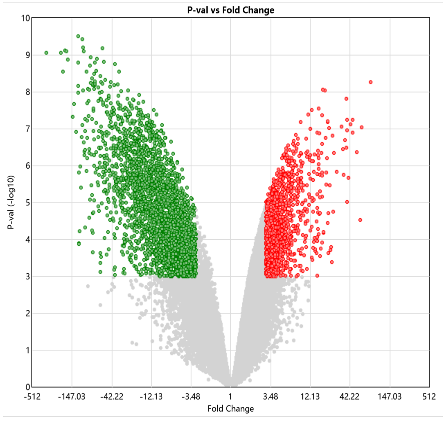

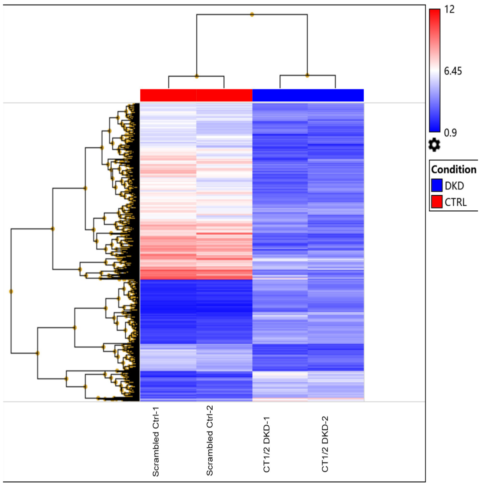

Supplement: Supplementary file 3 — Figure S1 [file 41389_2021_344_MOESM3_ESM.pdf]
