# Supplementary figures and images for: CtBP1/2 differentially regulate genomic stability and DNA repair pathway in high-grade serous ovarian cancer cell
Source: Oncogenesis. 2021 Jul 13;10(7):49. doi: 10.1038/s41389-021-00344-9 (PMC8275597; doi:10.1038/s41389-021-00344-9)

A

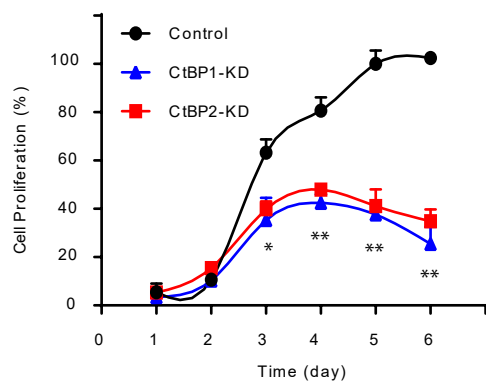

B

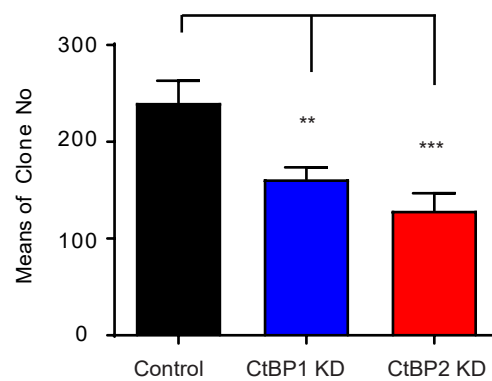

C

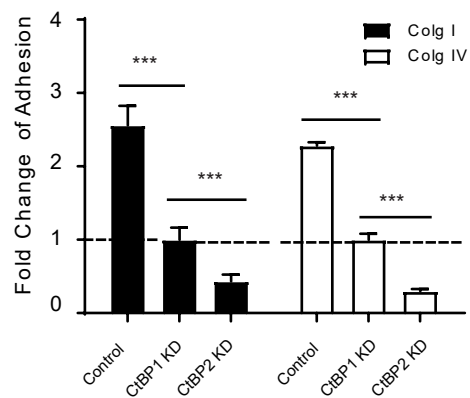

D

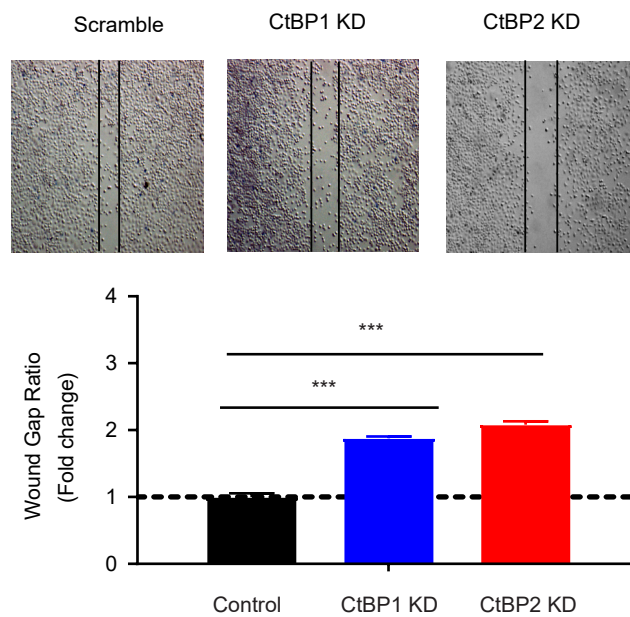

E

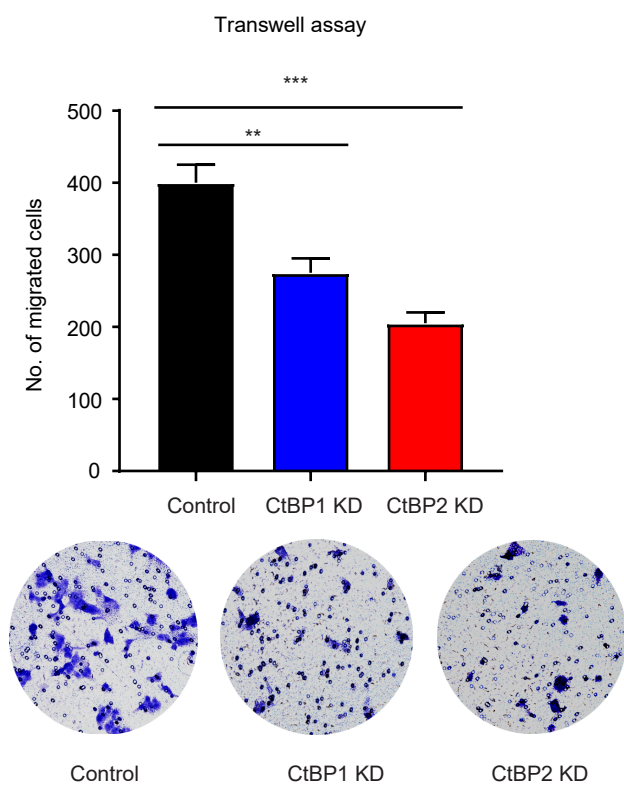

Supplement: Supplementary file 4 — Figure S2 [file 41389_2021_344_MOESM4_ESM.pdf]

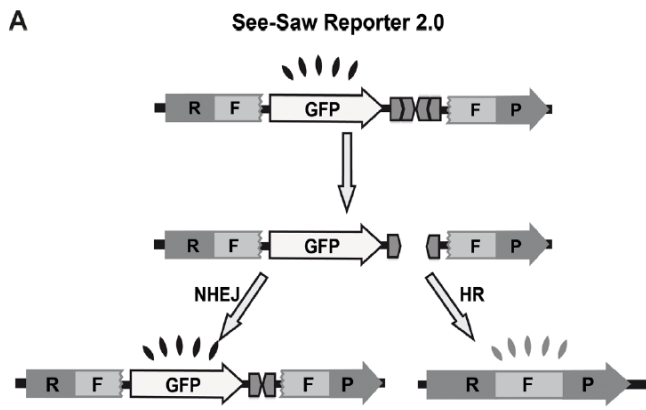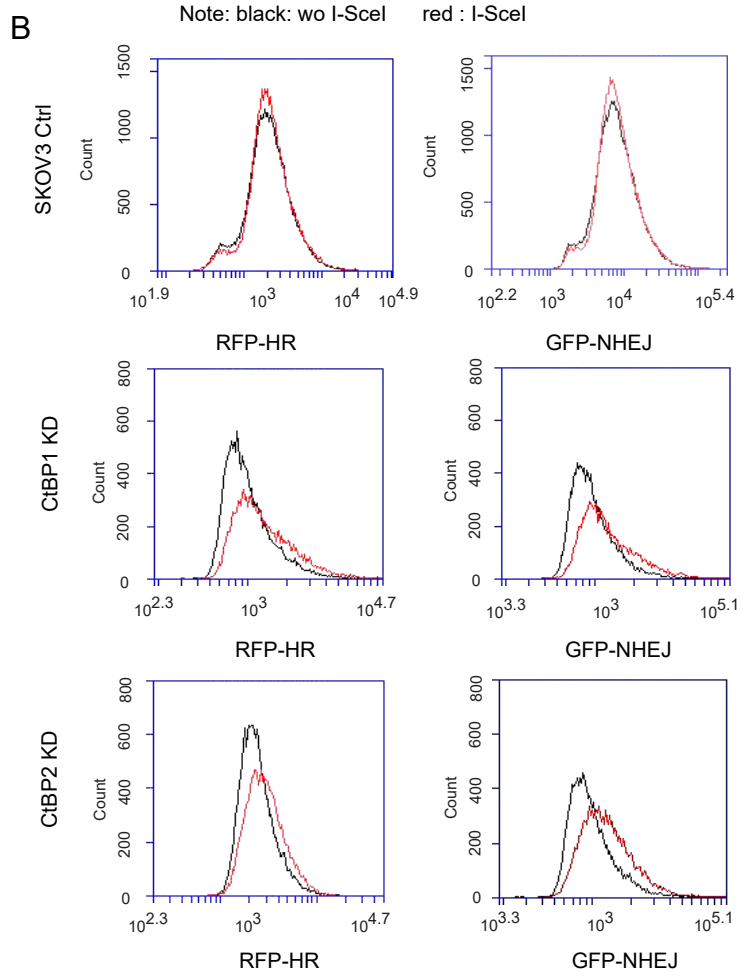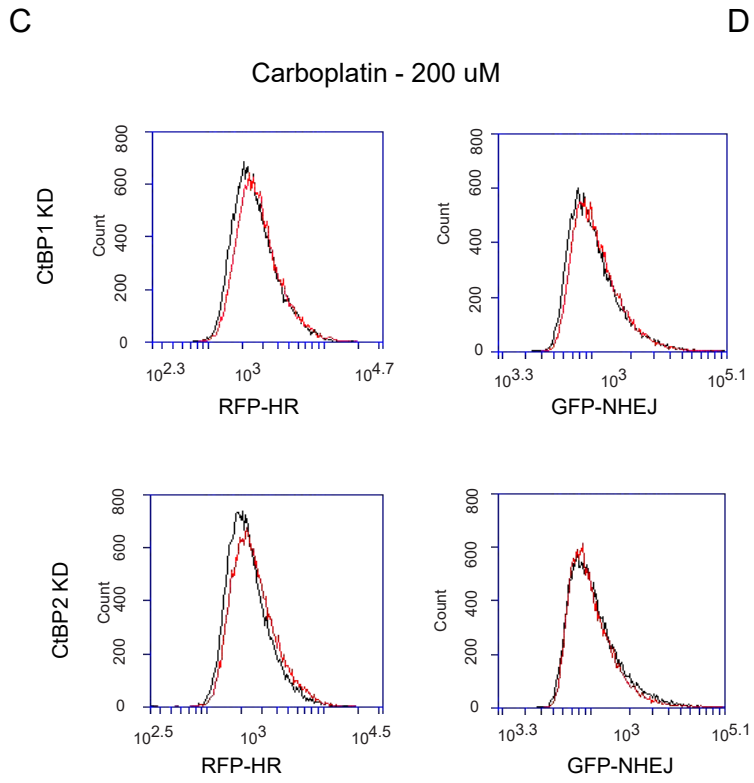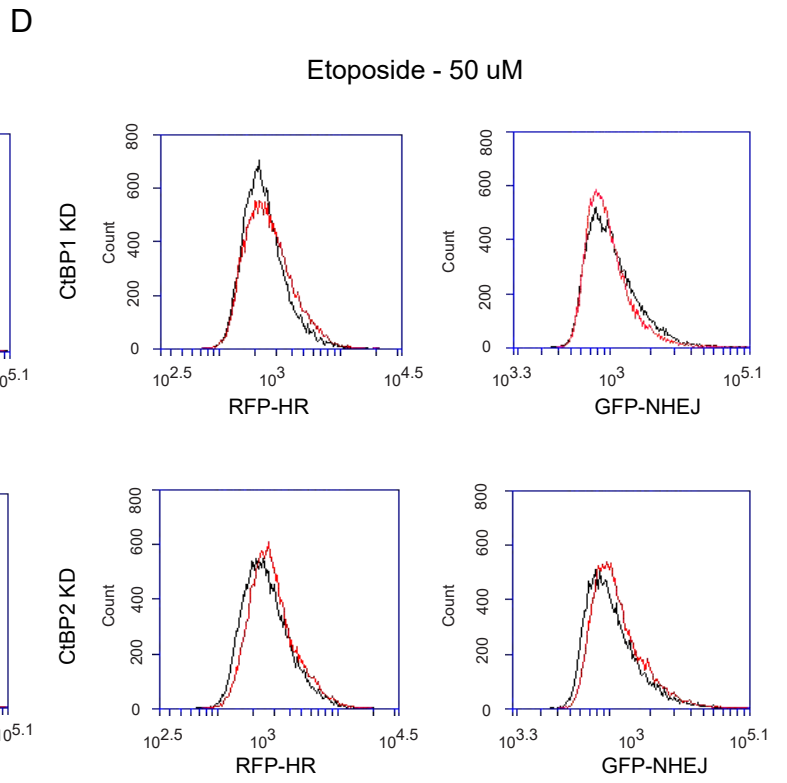

Supplement: Supplementary file 5 — Figure S3 [file 41389_2021_344_MOESM5_ESM.pdf]

A

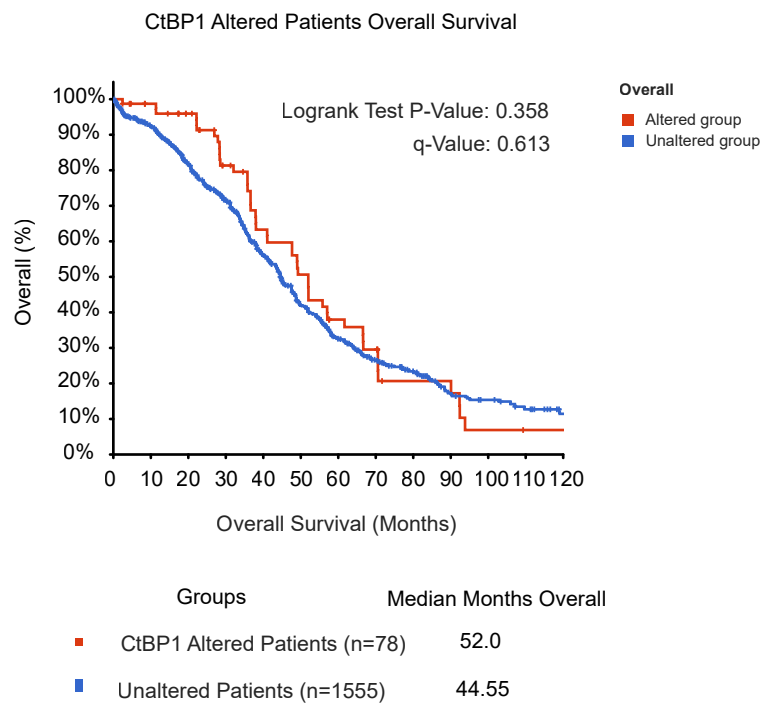

B

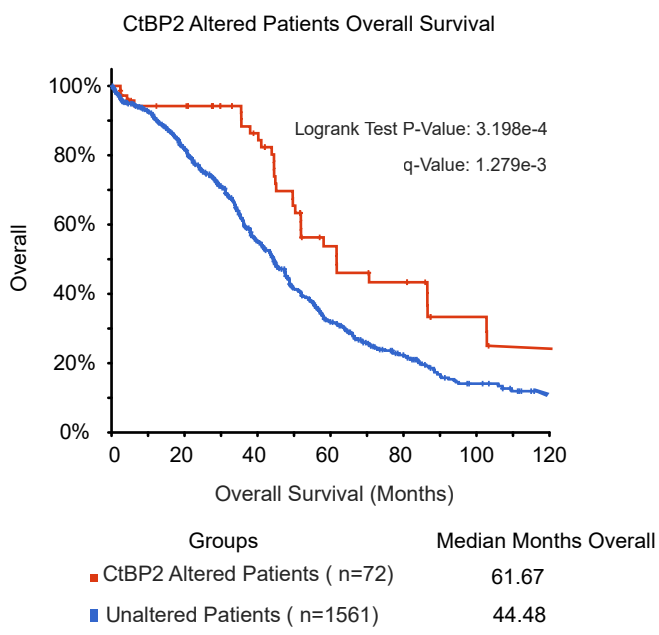

Supplement: Supplementary file 6 — Figure S4 [file 41389_2021_344_MOESM6_ESM.pdf]
