# Supplementary material for: CtBP1/2 differentially regulate genomic stability and DNA repair pathway in high-grade serous ovarian cancer cell
Source: Oncogenesis. 2021 Jul 13;10(7):49. doi: 10.1038/s41389-021-00344-9 (PMC8275597; doi:10.1038/s41389-021-00344-9)
Supplement: Supplementary file 7 — Table S1 [file 41389_2021_344_MOESM7_ESM.pdf]

Table. S1 The overlapped signficant changed genes among CtBP1/2 knockdown groups.

No.	Gene Symbol	Description
1	ABI1	abl-interactor 1
2	ADAM12	ADAM metallopeptidase domain 12
3	AFAP1L2	actin filament associated protein 1-like 2
4	ANKRD20A11P	ankyrin repeat domain 20 family, member A11, pseudogene
5	ARRB1	arrestin, beta 1
6	C16orf62	chromosome 16 open reading frame 62
7	C1DP3	C1D nuclear receptor corepressor pseudogene 3
8	C21orf59	chromosome 21 open reading frame 59
9	CA5B	carbonic anhydrase VB, mitochondrial
10	CACNA2D1	calcium channel, voltage-dependent, alpha 2/delta subunit 1
11	CMTM7	CKLF-like MARVEL transmembrane domain containing 7
12	COL4A2	collagen, type IV, alpha 2
13	CREB5	cAMP responsive element binding protein 5
14	CXCL1	chemokine (C-X-C motif) ligand 1 (melanoma growth stimulating activity, alpha)
15	CYP4F29P	cytochrome P450, family 4, subfamily F, polypeptide 29, pseudogene
16	ENTPD4	ectonucleoside triphosphate diphosphohydrolase 4
17	EPAS1	endothelial PAS domain protein 1
18	FAM131C	family with sequence similarity 131, member C
19	FNDC4	fibronectin type III domain containing 4
20	HCFC1R1	host cell factor C1 regulator 1 (XPO1 dependent)
21	HIST1H1B	histone cluster 1, H1b
22	HIST1H3J	histone cluster 1, H3j
23	HTATIP2	HIV-1 Tat interactive protein 2
24	IL15RA	interleukin 15 receptor, alpha
25	IL1R1	interleukin 1 receptor, type I
26	IL6	interleukin 6
27	ITGB3	integrin beta 3
28	KIF3C	kinesin family member 3C
29	KIN	Kin17 DNA and RNA binding protein
30	KRT17P2	keratin 17 pseudogene 2
31	LIMK1	LIM domain kinase 1
32	LOC102723462	uncharacterized LOC102723462
33	LRRN4	leucine rich repeat neuronal 4
34	MARK1	MAP/microtubule affinity-regulating kinase 1
35	MEST	mesoderm specific transcript
36	MT1B	metallothionein 1B

37	MT1F	metallothionein 1F
38	OAS3; OAS2	2-5-oligoadenylate synthetase 3; 2-5-oligoadenylate synthetase 2
39	ODC1	ornithine decarboxylase 1
40	PLLP	plasmolipin
41	PROS1	protein S (alpha)
42	PXDN	peroxidasin
43	RAI14	retinoic acid induced 14
44	REXO1	REX1, RNA exonuclease 1 homolog
45	ROR1	receptor tyrosine kinase-like orphan receptor 1
46	RPSA; RPSAP19	ribosomal protein SA; ribosomal protein SA pseudogene 19
47	RWDD1	RWD domain containing 1
48	SERPINE1	serpin peptidase inhibitor, clade E (nexin, plasminogen activator inhibitor type 1), member 1
49	SLC17A8	solute carrier family 17 (vesicular glutamate transporter), member 8
50	SLC4A3	solute carrier family 4 (anion exchanger), member 3
51	SVIL	supervillin
52	TFPI2	tissue factor pathway inhibitor 2
53	TGFBI	transforming growth factor, beta-induced, 68kDa
54	TMEM8A	transmembrane protein 8A
55	UCHL1	ubiquitin C-terminal hydrolase L1
56	ZNF485	zinc finger protein 485
