# Supplementary material for: CtBP1/2 differentially regulate genomic stability and DNA repair pathway in high-grade serous ovarian cancer cell
Source: Oncogenesis. 2021 Jul 13;10(7):49. doi: 10.1038/s41389-021-00344-9 (PMC8275597; doi:10.1038/s41389-021-00344-9)
Supplement: Supplementary file 8 — Table S2 [file 41389_2021_344_MOESM8_ESM.pdf]

Table S2. The Histogram comparisons of IdU labelled track between with or without Hu treatment among different groups.

**Cell lines**	**Control**	**Control**	**CtBP1-KD**	**CtBP1-KD**	**CtBP2-KD**	**CtBP2-KD**
Fiber labelled	IdU	IdU	IdU	IdU	IdU	IdU
Hu Treatment	without	with	without	with	without	with
Total number of values	463	368	486	467	564	323
Minimum	1.4790	0.8832	0.4921	0.4403	1.1240	0.4921
25% Percentile	3.9080	2.9680	1.3420	1.1890	3.5700	2.6470
**Median**	**7.0400**	**4.5340**	**1.7790**	**1.5590**	**5.3210**	**4.2140**
75% Percentile	12.4800	7.2490	2.6550	2.1730	8.5750	6.6300
Maximum	152.3000	58.7400	17.0800	8.4070	46.5000	24.1600
Mean	10.1300	5.9200	2.3530	1.7410	7.1760	5.2160
Std. Deviation	10.7500	5.2800	1.9220	0.9031	5.6050	3.5470
Std. Error	0.4998	0.2753	0.0872	0.0418	0.2360	0.1973
Lower 95% CI of mean	9.1520	5.3780	2.1810	1.6590	6.7120	4.8280
Upper 95% CI of mean	11.1200	6.4610	2.5240	1.8230	7.6400	5.6050
**P value**	**< 0.0001**		**< 0.0001**		**< 0.0001**	
Exact or approximate P value?	Gaussian Approximation		Gaussian Approximation		Gaussian Approximation	
P value summary	***		***		***	
Are medians signif. different? (P < 0.05)	**Yes**		**Yes**		**Yes**	
One- or two-tailed P value?	Two-tailed		Two-tailed		Two-tailed	
Sum of ranks in columns	271500	122300	271500	122300	271500	122300
Mann-Whitney U	70010		70010		70010	
