# Supplementary material for: CtBP1/2 differentially regulate genomic stability and DNA repair pathway in high-grade serous ovarian cancer cell
Source: Oncogenesis. 2021 Jul 13;10(7):49. doi: 10.1038/s41389-021-00344-9 (PMC8275597; doi:10.1038/s41389-021-00344-9)
Supplement: Supplementary file 9 — Table S3 [file 41389_2021_344_MOESM9_ESM.pdf]

Table S3. The Histogram comparisons of CldU labelled track between with or without Hu treatment among different groups.

**Cell lines**	**Control**	**Control**	**CtBP1-KD**	**CtBP1-KD**	**CtBP2-KD**	**CtBP2-KD**
Fiber labelled	CldU	CldU	CldU	CldU	CldU	CldU
Hu Treatment	with	without	with	without	with	without
Total number of values	464	389	550	466	607	382
Minimum	0.3522	0.4403	0.2202	0.2202	0.3108	0.3108
25% Percentile	2.0930	0.9868	0.9350	0.8832	1.2480	0.8832
**Median**	**3.1820**	**1.2870**	**1.2870**	**1.1030**	**1.7790**	**1.1030**
75% Percentile	5.2940	1.7790	1.8730	1.4120	2.7270	1.3420
Maximum	21.2300	27.7900	12.8000	3.1570	14.7200	5.0790
Mean	4.0330	1.7470	1.6640	1.1730	2.2640	1.1500
Std. Deviation	2.8260	1.9340	1.3870	0.4441	1.6610	0.5018
Std. Error	0.1312	0.0981	0.0591	0.0206	0.0674	0.0257
Lower 95% CI of mean	3.7760	1.5550	1.5480	1.1330	2.1320	1.1000
Upper 95% CI of mean	4.2910	1.9400	1.7800	1.2140	2.3970	1.2010
**P value**	**< 0.0001**		**< 0.0001**		**< 0.0001**	
Exact or approximate P value?	Gaussian Approximation		Gaussian Approximation		Gaussian Approximation	
P value summary	***		***		***	
Are medians signif. different? (P < 0.05)	Yes		Yes		Yes	
One- or two-tailed P value?	Two-tailed		Two-tailed		Two-tailed	
Sum of ranks in columns	260600	103600	308000	208600	365199	124356
Mann-Whitney U	27740		99810		51200	
