# Supplementary material for: CtBP1/2 differentially regulate genomic stability and DNA repair pathway in high-grade serous ovarian cancer cell
Source: Oncogenesis. 2021 Jul 13;10(7):49. doi: 10.1038/s41389-021-00344-9 (PMC8275597; doi:10.1038/s41389-021-00344-9)
Supplement: Supplementary file 10 — Table S4 [file 41389_2021_344_MOESM10_ESM.pdf]

Table. S4 The type of CtBP1/2 genetic alterations in serous ovairan cancer.

| Gene | Study | Sample ID | Cancer Type | Protein Change | Mutation Type | Copy # | COSMIC | Allele Freq (T) | # Mut in Sample |
|---|---|---|---|---|---|---|---|---|---|
| CtBP2 | Ovarian Serous Cystadenocarcinoma | TCGA-36-1578-01 | Serous Ovarian Cancer | K434Nfs*33 | frameshifts deletion | ShallowDel | | | 50 |
| CtBP2 | Ovarian Serous Cystadenocarcinoma | TCGA-36-1578-01 | High-Grade Serous Ovarian Cancer | K434Nfs*33 | frameshifts deletion | ShallowDel | | | 47 |
| CtBP2 | Ovarian Serous Cystadenocarcinoma | TCGA-24-2020-01 | Serous Ovarian Cancer | CTBP2-KCNMA1 | Fusion | Gain | | | |
| CtBP1 | Ovarian Serous Cystadenocarcinoma | TCGA-13-0887-01 | Serous Ovarian Cancer | P308H | Missense | Amp | 1 | | 81 |
| CtBP1 | Ovarian Serous Cystadenocarcinoma | TCGA-13-0887-01 | Serous Ovarian Cancer | P308H | Missense | Gain | 1 | 0.51 | 143 |
| CtBP1 | Ovarian Serous Cystadenocarcinoma | TCGA-13-0887-01 | High-Grade Serous Ovarian Cancer | P308H | Missense | Gain | 1 | | 80 |
| CtBP1 | Ovarian Serous Cystadenocarcinoma | TCGA-25-2391-01 | Serous Ovarian Cancer | LYAR-CTBP1 | Fusion | Amp | | | 74 |
