# Supplementary material for: CtBP1/2 differentially regulate genomic stability and DNA repair pathway in high-grade serous ovarian cancer cell
Source: Oncogenesis. 2021 Jul 13;10(7):49. doi: 10.1038/s41389-021-00344-9 (PMC8275597; doi:10.1038/s41389-021-00344-9)
Supplement: Supplementary file 11 — Table S5 [file 41389_2021_344_MOESM11_ESM.pdf]

Table S5. The SKOV3's typical alternated genes enriched in the CtBP2 genetic altered cases group.

Gene	Alternation type	Cytoband	Altered group	Unaltered group	Log Ratio	p-Value	q-Value	Enriched in
NOTCH1	deletion	9q34.3	8 (10.67%)	58 (3.64%)	1.55	7.97E-03	0.0354	CTBP2 Genetic Altered group
CDKN2AIPNL	deletion	5q31.1	4 (5.33%)	16 (1.00%)	2.41	0.0106	0.0431	CTBP2 Genetic Altered group
TP53AIP1	deletion	11q24.3	11 (14.67%)	65 (4.08%)	1.85	3.92E-04	4.52E-03	CTBP2 Genetic Altered group
TP53INP1	deletion	8q22.1	16 (21.33%)	133 (8.35%)	1.35	6.08E-04	6.12E-03	CTBP2 Genetic Altered group
TP53INP2	deletion	20q11.22	9 (12.00%)	49 (3.08%)	1.96	8.27E-04	7.47E-03	CTBP2 Genetic Altered group
TP53RK	deletion	20q13.12	10 (13.33%)	69 (4.33%)	1.62	2.15E-03	0.0142	CTBP2 Genetic Altered group
TP53I11	deletion	11p11.2	5 (6.67%)	18 (1.13%)	2.56	2.87E-03	0.0173	CTBP2 Genetic Altered group
TP53I3	deletion	2p23.3	5 (6.67%)	24 (1.51%)	2.15	8.18E-03	0.0358	CTBP2 Genetic Altered group
TP53TG3	deletion	16p11.2	6 (8.00%)	36 (2.26%)	1.82	9.74E-03	0.0403	CTBP2 Genetic Altered group
TP53TG3B	deletion	16p11.2	6 (8.00%)	36 (2.26%)	1.82	9.74E-03	0.0403	CTBP2 Genetic Altered group
TP53BP1	deletion	15q15.3	6 (8.00%)	41 (2.57%)	1.64	0.0167	0.0587	CTBP2 Genetic Altered group
TP53TG1	deletion	7q21.12	6 (8.00%)	43 (2.70%)	1.57	0.0202	0.0664	CTBP2 Genetic Altered group
TP53TG3C	deletion	16p11.2	4 (5.33%)	24 (1.51%)	1.82	0.034	0.0956	CTBP2 Genetic Altered group
TP53TG3D	deletion	16p11.2	4 (5.33%)	24 (1.51%)	1.82	0.034	0.0956	CTBP2 Genetic Altered group
TP53I13	deletion	17q11.2	3 (4.00%)	14 (0.88%)	2.19	0.0377	0.103	CTBP2 Genetic Altered group
CDKN2AIP	deletion	4q35.1	5 (6.67%)	38 (2.39%)	1.48	0.0407	0.109	CTBP2 Genetic Altered group
RB1	deletion	13q14.2	12 (16.00%)	166 (10.42%)	0.62	0.0949	0.197	CTBP2 Genetic Altered group
ERBB2	Amplication	17q12	4 (5.33%)	57 (3.58%)	0.58	0.2930	0.428	CTBP2 Genetic Altered group
NOTCH2	deletion	1p12	10 (13.33%)	117 (7.34%)	0.86	0.0536	0.133	CTBP2 Genetic Altered group
MLH1	deletion	3p22.2	4 (5.33%)	29 (1.82%)	1.55	0.0574	0.14	CTBP2 Genetic Altered group
